# Engineering half-metallicity in wurtzite Zn_1−2*x*_Mn_*x*_A_*x*_S (A = Mo, Ni) for enhanced optoelectronic and thermoelectric performance: a DFT study

**DOI:** 10.1039/d5ra09710j

**Published:** 2026-01-06

**Authors:** W. Amghar, A. Fakhim Lamrani, K. Sadki, F. E. EL Mabchour, E. Maskar, A. El hat, S. Otmani, R. Ahl Laamara

**Affiliations:** a LPHE-Modeling and Simulation, Faculty of Sciences, Mohammed V University in Rabat Rabat Morocco fakhim@ik.me fakhim@ens.um5.ac.ma; b ENS-Rabat Physics Department, Mohammed V University in Rabat B. P. 5118 Morocco f.elmabchour@um5r.ac.ma; c Centre of Physics and Mathematics, CPM, Faculty of Sciences, Mohammed V University in Rabat Rabat Morocco

## Abstract

The electronic, magnetic, optical, and thermoelectric properties of the artificially engineered (Mn, Mo) and (Mn, Ni) codoped ZnS systems have been investigated using density functional theory (DFT) within the Wien2K Package. Calculations were carried out using both the generalized gradient approximation (GGA) and the modified Becke–Johnson (mBJ) potential to ensure reliable electronic and magnetic descriptions. The double codoping of ZnS with (Mn, Mo) and (Mn, Ni) leads to the formation of an artificial half-metallic material, where both parallel and antiparallel spin configurations converge toward a ferromagnetic solution. However, the most stable phase corresponds to a ferrimagnetic configuration. The half-metallic character originates from strong p–d hybridization between transition-metal orbitals, which plays a crucial role in determining the material's multifunctional properties. The optical response exhibits a noticeable redshift in the absorption edge and distinct plasmonic structures, demonstrating the potential of such half-metallic systems for optoelectronic and photonic applications. Furthermore, thermoelectric calculations reveal that (Mn, Mo) codoping induces a p-type Seebeck coefficient with a figure of merit (*ZT*) ≈ of 1.2. In contrast, (Mn, Ni) codoping exhibits *n*-type behaviour with an enhanced *ZT* ≈ 1.6. These results highlight that (Zn_1−2*x*_Mn_*x*_A_*x*_S) (A = Mo, Ni) represents a promising artificial half-metallic material with significant potential for multifunctional spintronic, optoelectronic, and thermoelectric applications.

## Abstract 2

DFT calculations reveal that (Mn, Mo) and (Mn, Ni) codoped ZnS exhibit artificial half-metallicity driven by p–d hybridization, ferrimagnetic ground-state stabilization, redshifted optical response, and enhanced thermoelectric performance for multifunctional applications.

## Graphical abstract

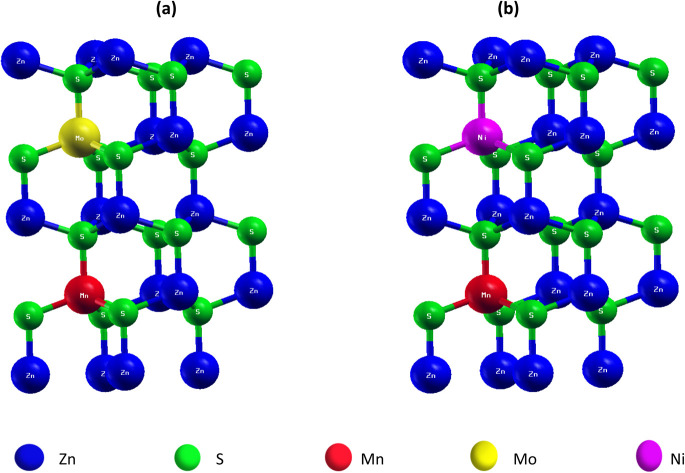

## 1. Introduction

The discovery of diluted magnetic semiconductors (DMSs) has opened new avenues for investigating magnetic phenomena in materials with relatively simple band structures while retaining favourable magneto-optical and charge-transport properties. In this context, II–VI compound semiconductors have attracted sustained attention, particularly in nanocrystalline and quantum-dot forms, owing to their tunable optical emission and compatibility with low-cost colloidal synthesis routes.^1–5^ Zinc sulfide (ZnS), a representative II–VI semiconductor, is characterized by a wide band gap of approximately 3.7 eV^6,7^ and relatively large exciton binding energy (40 meV) of zinc sulfide (ZnS),^8,9^ making it a versatile material for photonic and optoelectronic applications, especially in the violet and blue spectral regions.^10,11^ ZnS crystallizes predominantly in the cubic zinc blende structure under ambient conditions, while the hexagonal wurtzite phase can be stabilized at elevated temperatures.^12^ In addition to its favourable electronic properties, ZnS is non-toxic, inexpensive, and abundant, further enhancing its technological appeal.^13^ Doping is a widely adopted strategy to tailor the optical, electronic, and magnetic properties of semiconductors by introducing localized states or impurity-derived energy levels within the band gap. In systems containing magnetic dopants, such modifications may give rise to intriguing magnetic, magneto-optical, and spin-dependent transport phenomena, thereby expanding their functional scope.^10,14–16^ Both experimental and theoretical studies have reported room-temperature ferromagnetism and spin-polarized electronic states in transition-metal-doped ZnS, including Fe-, Co-, Ni-, V-, and Ti-doped systems.^17–21^ Experimental work by Amaranatha Reddy *et al.* demonstrated that light Cr doping in ZnS nanoparticles induces intrinsic ferromagnetic ordering at room temperature.^22^ From an optical perspective, impurity incorporation also enables effective tuning of band-gap energies and photoluminescence characteristics. For example, ZnS : Te, ZnS : Cu^2+^, ZnS : Ni^2+^, and ZnS : Mn^2+^ nanocrystals have been reported to emit across a broad range of visible wavelengths.^23–26^ Among these, Mn-doped ZnS has been extensively studied, with both experimental and theoretical investigations confirming robust ferromagnetic ordering at ambient conditions.^10,27^ However, Mn monodoping alone does not generally yield a fully spin-polarized electronic structure, which limits its potential for spintronic applications. Co-doping with an additional transition metal has therefore been proposed as a possible route to enhance spin polarization or induce spin-selective transport features, although this strategy has received comparatively limited attention. Furthermore, most previous theoretical studies have focused on the cubic zinc blende phase, while the hexagonal wurtzite structure of ZnS remains significantly less explored in this context.^28,29^ Beyond spin-dependent phenomena, ZnS-based materials have also been considered for thermoelectric applications due to the tunability of their electronic and transport properties *via* doping. Thermoelectric behaviour has been widely investigated in oxides, chalcogenides, and related semiconductors, particularly in the context of waste-heat recovery and energy conversion.^30–34^ Doped semiconductors, including ZnS and its derivatives, have attracted interest owing to the possibility of modulating carrier concentration and transport coefficients through controlled impurity incorporation.^35–38^ Nevertheless, systematic studies of thermoelectric trends in diluted magnetic semiconductors, especially ZnS-based systems, remain relatively scarce. In particular, the interplay between magnetic ordering, electronic structure, and charge transport has not yet been fully clarified, and the integration of spin-dependent, optoelectronic, and transport functionalities within a single ZnS-based platform remains largely unexplored.^39–41^

### 1.1 Motivation and experimental relevance

Although transition-metal doping of ZnS (*e.g.*, Mn-, Ni-, or Mo-doped ZnS) has been widely demonstrated experimentally, the cooperative effects arising from transition-metal co-doping have received far less attention. Co-doping offers additional degrees of freedom by simultaneously modifying the electronic states associated with multiple dopants, which may alter spin polarization, band-edge characteristics, or defect-level distributions beyond what is achievable in single-dopant systems. Recent experimental studies have reported the successful synthesis of Mn–Ni co-doped ZnS nanocrystals using wet-chemical and precipitation methods, confirming the structural stability and experimental feasibility of multi-dopant incorporation.^42,43^ In addition, Ni-doped ZnS nanoparticles with controlled morphology and optical activity have been synthesized *via* hydrothermal and solvothermal routes.^44^ Further supporting the practical relevance of transition-metal incorporation in ZnS.

In this context, first-principles calculations can provide valuable insights into the underlying electronic and magnetic mechanisms governing co-doped ZnS systems. In the present work, we investigate the influence of Mn–Mo and Mn–Ni co-doping on the electronic, magnetic, optical, and thermoelectric properties of wurtzite ZnS. The primary objective is to elucidate how co-doping modifies spin polarization, band-edge features, and transport trends, rather than to predict absolute device-level performance. Electronic structure calculations are carried out within density functional theory (DFT) using both the generalized gradient approximation of Perdew–Burke–Ernzerhof (GGA-PBE) ^45^ and the modified Becke–Johnson (mBJ) exchange potential,^46^ which provides an improved description of band-gap characteristics. All calculations are performed using the WIEN2k package,^47,48^ based on the full-potential linearized augmented plane-wave (FP-LAPW) method.^49^ The remainder of this paper is organized as follows. Section 2 describes the computational methodology, while Section 3 presents and discusses the structural, electronic, magnetic, optical, and thermoelectric results. The main conclusions and perspectives are summarized in Section 4.

## 2. Computational model and method

Structural and electronic properties were investigated using density functional theory (DFT) within the full-potential linearized augmented plane wave (FP-LAPW) method as implemented in WIEN2k. The wurtzite ZnS host (space group *P*6_3_*mc*) was modelled using the experimental lattice parameters *a* = *b* = 3.82 Å and *c* = 6.26 Å. Zn and S atoms occupy the Wyckoff 2b positions (1/3, 2/3, *u*) and (1/3, 2/3, *v*).^50^ Exchange–correlation effects were described using the generalized gradient approximation (GGA-PBE) and the modified Becke–Johnson (mBJ) potential, with the mBJ parameter *c* determined self-consistently. The basis-set size was controlled using RMT × *K*_max_ = 7, with energy and charge convergence criteria of 10^−6^ Ry and 10^−6^ e, respectively. Atomic forces were relaxed below 0.1 mRy au^−1^. A 2 × 2 × 2 Zn_16_S_16_ supercell was constructed to model the codoping concentration *x* = 0.0625 in Zn_1−2*x*_Mn_*x*_A_*x*_S (A = Mo, Ni). Brillouin-zone integrations employed a Monkhorst–Pack 8 × 8 × 4 *k*-mesh, corresponding to ≈300 *k*-points in the irreducible Brillouin zone; this mesh was used consistently for all self-consistent electronic, optical, and transport calculations.

For codoped systems, spin–orbit coupling (SOC) was included self-consistently to account for the relativistic. Ferromagnetic (FM) and antiferromagnetic (AFM) configurations were examined, and the energy difference Δ*E* = *E*_AFM_ − *E*_FM_ was evaluated to determine magnetic stability. The AFM state was consistently found to be lower in energy, confirming antiferromagnetic ground-state stabilization.

Thermoelectric transport properties were computed using BoltzTraP, employing the same *k*-mesh and the constant relaxation-time approximation with *τ* = 10^−14^ s. Electrical conductivity, Seebeck coefficient, and electronic thermal contributions were obtained within the semiclassical Boltzmann framework. We note that transport coefficients are sensitive to *k*-point sampling. However, the present work employed the 8 × 8 × 4 mesh (≈300 IBZ *k*-points) consistently. Further convergence tests with denser meshes could be performed to achieve fully converged *κ*_e_ and *ZT* values.

## 3. Results and discussions

**Fig. 1:**
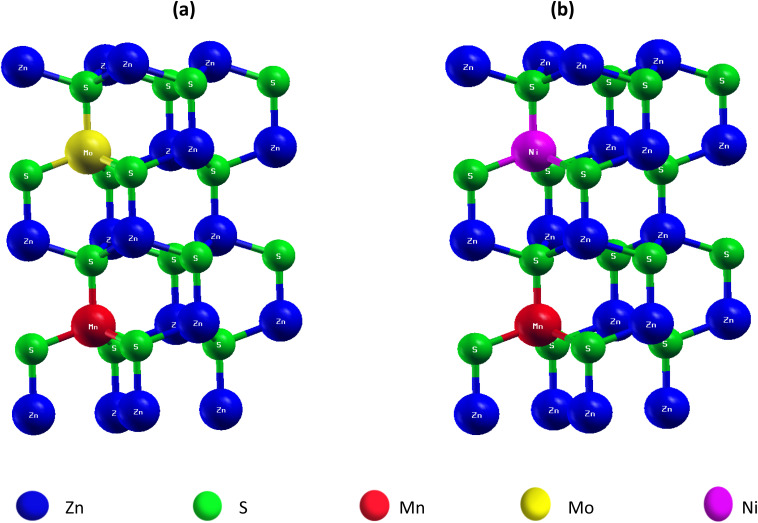
Crystal structures of codoped ZnS supercells. (a) Mn/Mo co-doped ZnS (Zn_1−2*x*_Mn_*x*_Mo_*x*_S), (b) Mn/Ni co-doped ZnS (Zn_1−2*x*_Mn_*x*_Ni_*x*_S), (*x* = 0.0625).

The crystal structures of the codoped ZnS supercells are shown in Fig. 1. Panel (a) depicts the Mn/Mo co-doped configuration (Zn_1−2*x*_Mn_*x*_Mo_*x*_S), while panel (b) shows the Mn/Ni co-doped configuration (Zn_1−2*x*_Mn_*x*_Ni_*x*_S). The figure highlights the positions of the dopant atoms relative to the Zn and S lattice sites and was generated using XCrySDen, providing a clear visualization of the dopant arrangements within the ZnS lattice.

Having presented the optimized structures, the thermodynamic stability of single- and co-doped ZnS was evaluated by calculating the formation energies (*E*_f_). This quantity measures the energetic favourability of substituting Zn atoms with dopants in the ZnS lattice. For a general doped configuration, *E*_f_ is defined as:^51^

1 *E*_f_ (Zn_N–nMn–nA_Mn_nMn_A_nA_S_N_) = *E*_tot_(doped) − *E*_tot_(pristine) + (*n*_Mn_ + *n*_A_) µ(Zn) − *n*_Mn_ µ(Mn) − *n*_A_µ(A)

Where *E*_tot_(doped) and *E*_tot_(pristine) are the total energies of the doped and pristine 32-atom ZnS supercells, respectively. *n*_Mn_ and *n*_A_ denote the number of Zn atoms replaced by Mn and the secondary dopant A (Ni or Mo), while µ(Zn), µ(Mn), and µ(A) are the chemical potentials of the elements in their most stable phases. The calculated formation energies for both single- and co-doped configurations are summarized in Table 1. All values are negative, confirming that substitution of Zn atoms by Mn, Ni, or Mo is thermodynamically favourable. Among the co-doped systems, Mn/Ni shows the most negative formation energy per dopant, indicating enhanced stability relative to single-doped and Mn/Mo co-doped systems.

**Table 1:** Thermodynamic stability and magnetic interactions in Mn/Ni and Mn/Mo co-doped ZnS: A first-principles study. Energies per dopant are normalized to the number of substituted atoms. All calculations correspond to AFM configurations; chemical potentials are taken from elemental reference states

Supercell composition	Doping type	(*E*_f_) (eV)	(*E*_f_) per dopant (eV)	Δ*E* = *E*_FM_ – *E*_AFM_ (meV)
Zn_15_Mn_1_S_16_	Mn-doped	−30.981	−30.981	—
Zn_15_Mo_1_S_16_	Mo-doped	−24.583	−24.583	—
Zn_15_Ni_1_S_16_	Ni-doped	−26.047	−26.047	—
Zn_14_Mn_1_Mo_1_S_16_	Mn/Mo co-doped	−27.706	−13.853	12.3
Zn_14_Mn_1_Ni_1_S_16_	Mn/Ni co-doped	−29.156	−14.578	8.7

The magnetic ground state of all co-doped configurations is antiferromagnetic (AFM). The magnetic stability was evaluated through the energy difference Δ*E* = *E*_FM_ − *E*_AFM_. For the Mn/Mo codoped supercell, Δ*E* = 12.3 meV per supercell, while for Mn/Ni co-doping, Δ*E* = 8.7 meV per supercell. These small and positive Δ*E* values confirm that the AFM ordering is energetically preferred over FM.

The electronic structure remains essentially unchanged by the magnetic configuration, with all co-doped systems preserving a semiconducting or half-metallic nature. Overall, codoping enhances both the structural and magnetic stability of ZnS, highlighting its potential for experimental synthesis and spintronic applications.

### 3.1 Electronic and magnetic properties

**Fig. 2:**
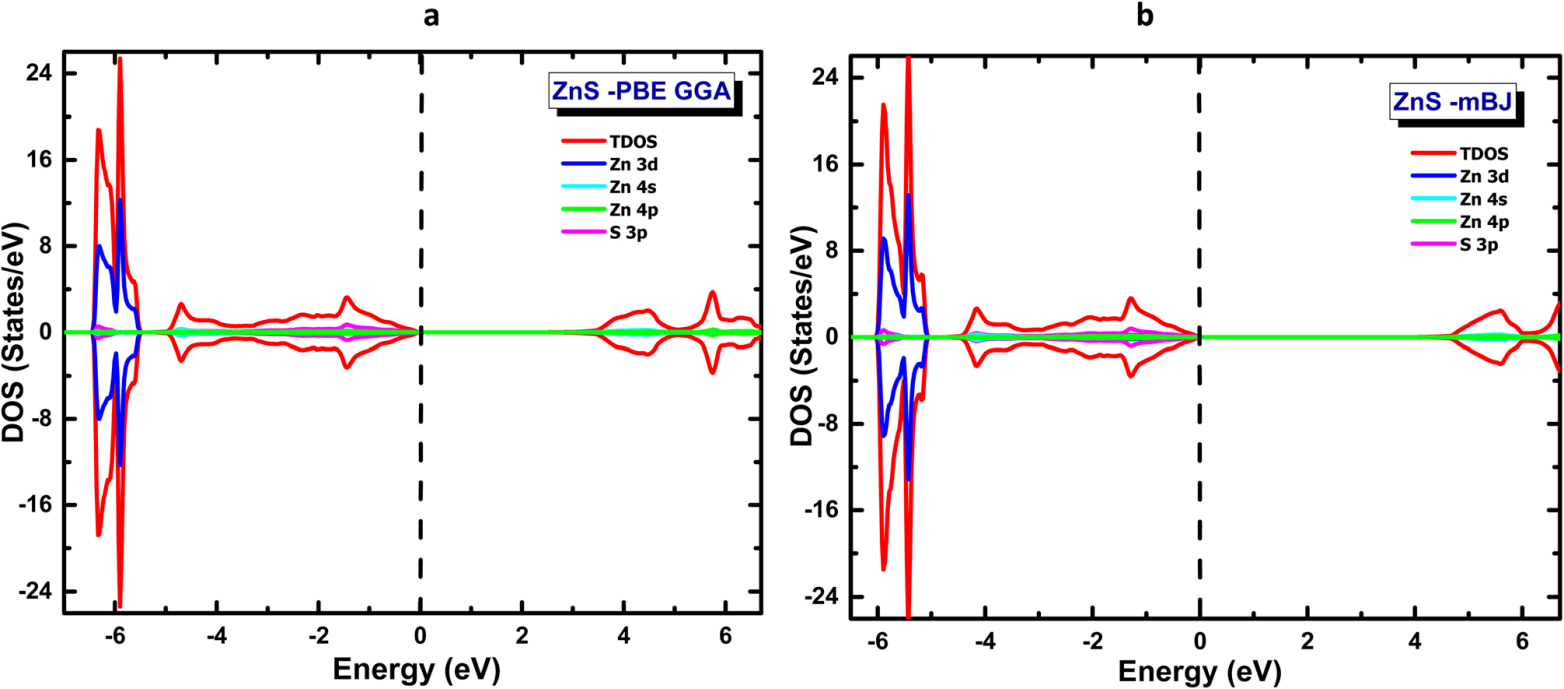
Total and partial density of states of pristine ZnS calculated using (a) GGA and (b) mBJ approaches.

The modified Becke-Johnson semi-local potential (mBJ) has been employed to enhance bandgap accuracy and more accurately characterize electronic properties. The equation that follows expresses this:^52^2
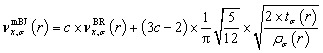
where the Becke–Roussel potential is denoted by *ν*_*x*,*σ*_^BR^ (*r*), the spin *σ* electron density by *ρ*_*σ*_(*r*), and the associated kinetic energy density by *t*_*σ*_(*r*). An enhanced description of local exchange interactions is provided by the automatic determination of the factor *c* from the gradient of the electron density. This approach allows for more precise calculation of electrical characteristics, especially in semiconductors, when compared to GGA approximations. Given the foregoing, the GGA and mBJ methods have been used to compute the total (TDOS) and partial (PDOS) density of states of ZnS in its pure form, as shown in Fig. 2a and b. The valence band (BV) under the GGA approximation is mostly between ∼ −6 eV and the Fermi level (0 eV) and is primarily dominated by sulfur 3p orbitals (S-3p), with a minor contribution from lower-localized zinc 3d orbitals (Zn-3d), which are located between −8 and −10 eV. The 4 s and 4p zinc orbitals make up the majority of the conduction band (BC), which is located above the Fermi level and is typical of II–VI semiconductors. The measured electronic gap value of about 2.16 eV is still less than the experimental value of about 3.8 eV, demonstrating GGA's well-known propensity to underestimate the bandgap energy.

With a gap opening of about 3.78 eV, which is substantially closer to the reported experimental values, the application of the mBJ potential, on the other hand, significantly improves this prediction. Better electronic localization is reflected by a shift in density peaks towards lower energies, especially in the conduction band, even though the nature of the electronic states is largely unchanged. Thus, the two approaches' comparison validates that mBJ is more effective at accurately characterizing ZnS electronic properties, which is consistent with experimental findings reported in the literature (see Table 2).

**Table 2:** Band gap (*E*_g_) values of wurtzite ZnS: experimental and theoretical comparison

References	Methods	*E*_g_ (eV)
Present work (GGA-PBE)	DFT (GGA-PBE)	2.16
Present work (mBJ)	DFT (mBJ)	3.78
Sharma & Mishra (2019) ref. 9	DFT (GGA- PBE)	2.20
D'Amico *et al.* (2017),ref. 53	DFT (GGA + U)	3.25
SWNT study (2019) ref. 54	DFT (HSE06)	3.51
Karazhanov *et al.* (2007) ref. 55	DFT (LDA)	1.90
Hussain, S. *et al.* (2019) ref. 56	DFT (mBJ)	3.70
Adachi, S. (1999) experimental data. ref 57	Optical measurement	3.80

**Fig. 3:**
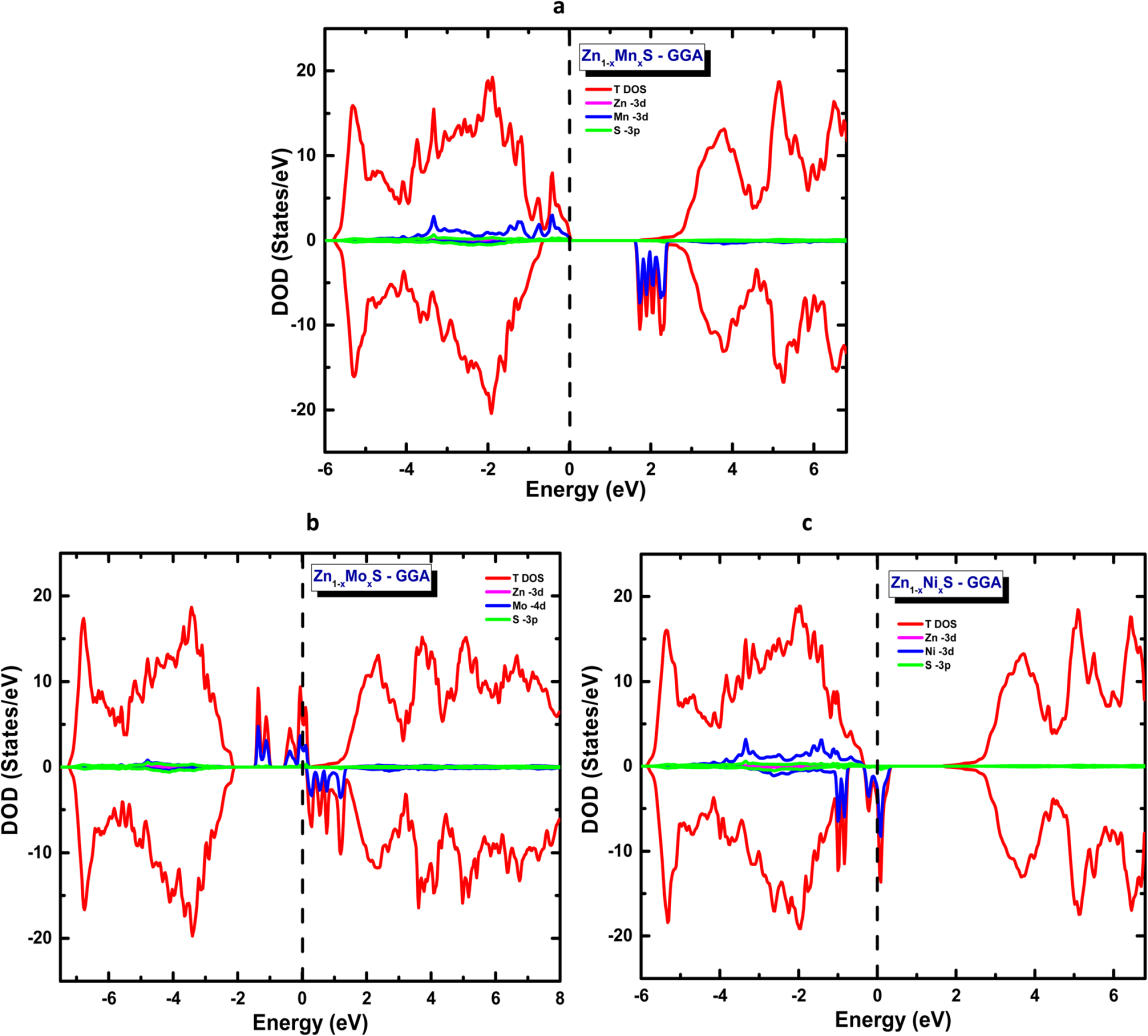
Total partial density of states of ZnS doped with (a) Mn, (b) Mo, and (c) Ni at *x* = 0.0625.

In continuation of our work, and before discussing the codoping of ZnS in the wurtzite structure, we first investigated the individual doping of each atom to assess its intrinsic behaviour within the host semiconductor matrix. The Mn-doped ZnS system, calculated within the framework of the generalized gradient approximation (GGA), is illustrated in Fig. 3a. The distribution of the Mn 3d orbitals shows that the majority-spin states are fully occupied up to the Fermi level *E*_f_, corresponding to the top of the valence band, while the minority-spin states remain unoccupied near the conduction band minimum. The evident asymmetry between the spin-up and spin-down channels reflects partial spin polarization, originating from the strong hybridization between Mn 3d and S 3p orbitals. The PDOS analysis of Zn_1−x_Mn_*x*_S (*x* = 00.625) confirms that Mn occurs in a divalent state (Mn^2+^), consistent with calculated total and local magnetic moments of 5 µB and 4.6 µB, respectively. Furthermore, our theoretical results indicate a reduction in the optical band gap following Mn incorporation, in good agreement with previous experimental observations (Gogoi *et al.*, 2010; El-Naggar *et al.*, 2023).^27,58^

Similarly, Fig. 3b presents the case of ZnS doped with molybdenum (Mo). The PDOS analysis reveals a ferromagnetic configuration with 100% spin polarization of the majority spin states of Mo 4d orbitals. The minority spin states are empty, leading to a total magnetic moment of 4 µB, while the partial moment of the Mo 4d orbitals is 2.4 µB. This value is slightly underestimated due to the GGA approximation. This data confirms that molybdenum adopts a tetravalent oxidation state (Mo^4+^) within the ZnS lattice. The calculated band gap in the spin-down channel is about 2.3 eV (Table 3), in good agreement with the experimental observations reported by Abdelhak Jrad *et al.*^59^

**Table 3:** Electronic and magnetic properties of Zn_1−*x*_M_*x*_S (M = Mn, Mo, Ni) (M = Mn, Mo, Ni) calculated within the GGA approach. Reported quantities include the band gap *E*_g_, Fermi energy *E*_f_ total magnetic moment *M*_tot_, and the local magnetic moment on the dopant atom

Property	Zn_1−*x*_Mn_*x*_S (GGA)	Zn_1−*x*_Mo_*x*_S (GGA)	Zn_1−*x*_Ni_*x*_S (GGA)
Band gap *E*_g_ (eV)	2.37	2.32	2.15
Fermi energy *E*_f_ (Ry)	0.2784	0.3982	0.2795
Total magnetic moment *M*_tot_ (µB)	5.000	4.001	2.000
Local moment on dopant (µB)	4.598	2.336	1.298

Finally, Fig. 3c illustrates ZnS doped with nickel Zn_1−*x*_Ni_*x*_S (*x* = 0.0625), which displays half-metallic behaviour with 100% spin polarization of the minority-spin, while the majority-spin states are fully occupied. The total magnetic moment of the system is 2 µB, and the partial moment on the Ni atom is 1.3 µB. This reduction stems from the increased filling of the Ni 3d orbitals, resulting in lower magnetic polarization at the Ni site. The band gap is approximately 2.15 eV (Table 3), in good agreement with previous theoretical and experimental reports.^9^ This analysis provides insight into the potential behavior of these dopants in a co-doped wurtzite ZnS matrix.

**Fig. 4:**
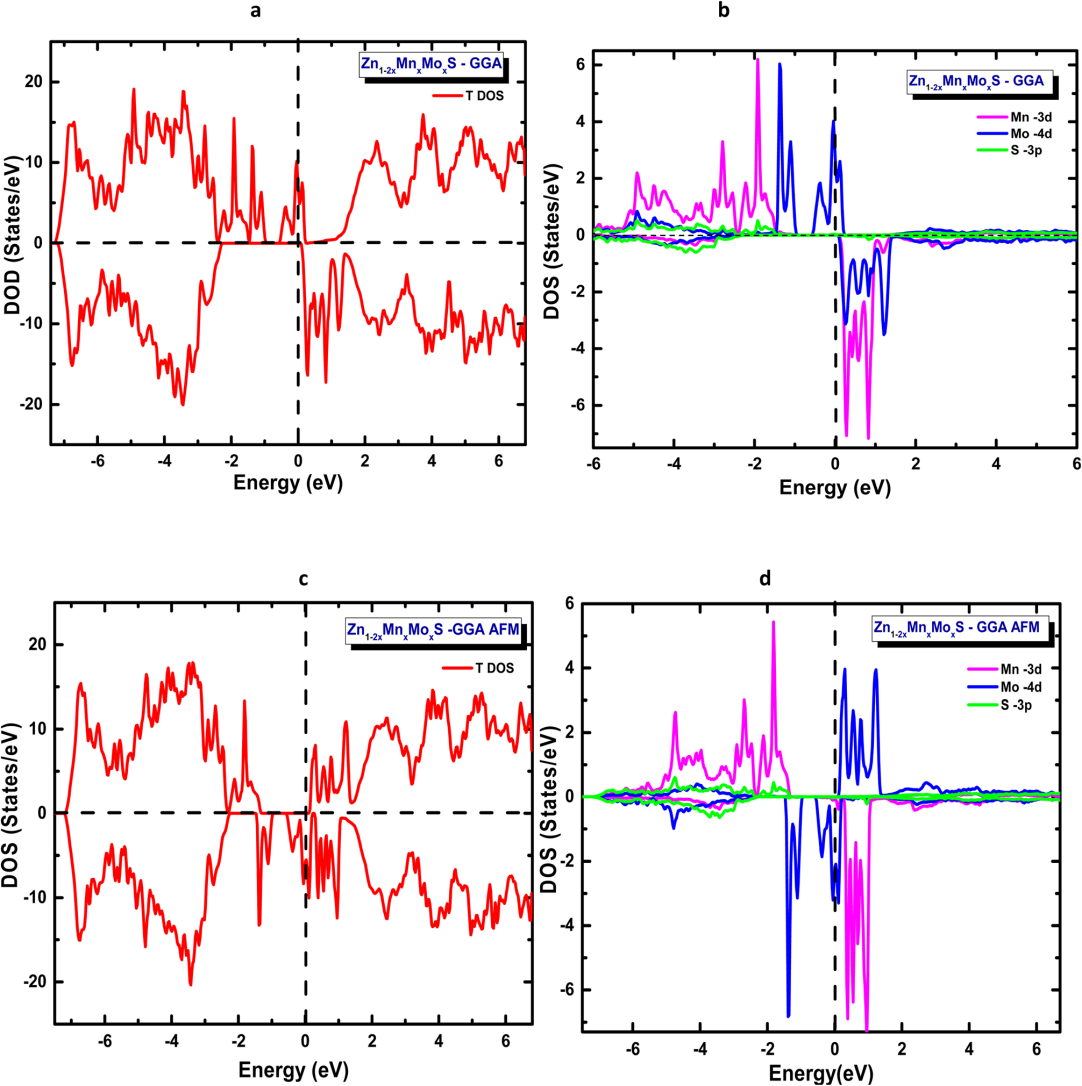
GGA-calculated total and partial density of states for ZnS codoped with Mn and Mo: (a) total DOS (FM), (b) partial DOS (FM), (c) total DOS (AFM), and (d) partial DOS (AFM).

**Fig. 5:**
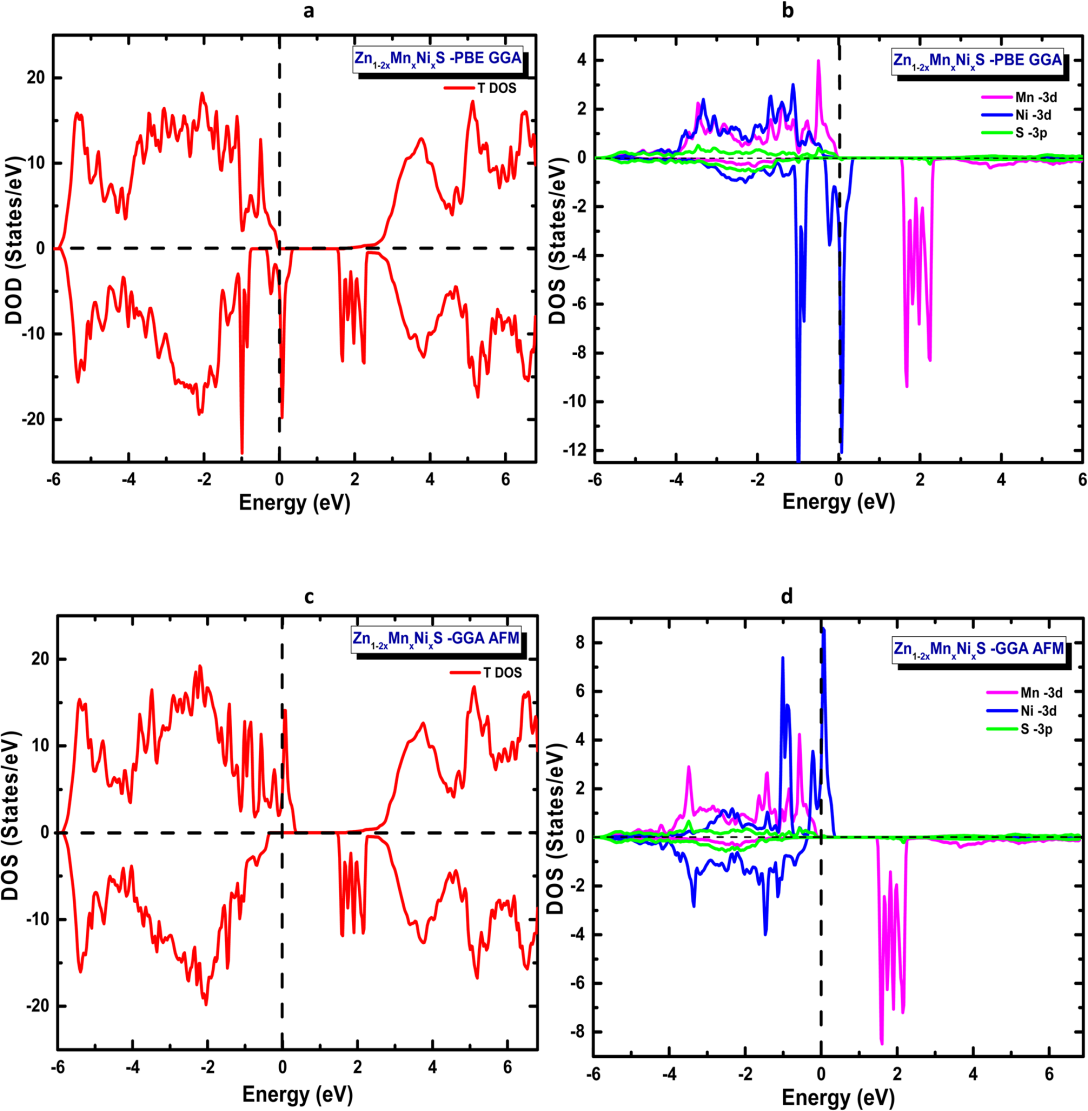
GGA-calculated total and partial density of states for ZnS codoped with Mn and Ni: (a) total DOS (FM), (b) partial DOS (FM), (c) total DOS (AFM) and (d) partial DOS (AFM).

In the codoping study of Zn_1−2*x*_Mn_*x*_A_*x*_S (A = Mo, Ni) at a concentration of *x* = 6.25%, the total energies corresponding to both spin configurations were first evaluated within the framework of the generalized gradient approximation (GGA) to identify the most stable magnetic ground state. The obtained results, summarized in Table 4, reveal that for both artificial compounds Zn_1−2*x*_Mn_*x*_Mo_*x*_S and Zn_1−2*x*_Mn_*x*_Ni_*x*_S the antiparallel spin alignment is energetically favoured and constitutes the magnetic ground state. This antiparallel coupling between inequivalent magnetic sublattices leads to a ferrimagnetic ground state, characterized by a non-zero net magnetic moment per formula unit. Accordingly, all electronic properties reported in Table 4, including the band gap, Fermi level, and magnetic moments, are evaluated within this configuration. The electronic structures exhibit a clear half-metallic character, as shown in Fig. 4 and 5, which present the total and partial densities of states for the two systems.

**Table 4:** Electronic and magnetic properties of codoped doped Zn_1−2*x*_Mn_*x*_A_*x*_S (A = Mo, Ni) calculated within the GGA and mBJ approaches. All reported quantities correspond to the antiferromagnetic (AFM) ground state. Listed properties include the band gap *E*_g_, the Fermi energy *E*_f_, the total magnetic moment *M*_tot_, and the local magnetic moments on the dopant atoms. Negative values indicate antiparallel spin alignment in the AFM configuration

Property	Mn/Mo (GGA)	Mn/Mo (mBJ)	Mn/Ni (GGA)	Mn/Ni (mBJ)
Band gap *E*_g_ (eV)	1.56	2.45	1.79	3.31
Fermi energy *E*_Fermi_ (Ry)	0.3985	0.4014	0.2842	0.2940
Total moment *M*_tot_ (µB)	0.9997	0.9997	3.0002	3.0002
Local *M*_Mn_ (µB)	4.177	4.201	4.190	4.353
Local *M*_Mo_ (µB)	−2.322	2.340	—	—
Local *M*_Ni_ (µB)	—	—	−1.290	−1.687

In this framework, both the generalized gradient approximation (GGA) and the modified Becke–Johnson (mBJ) potential were employed to achieve a more accurate and comprehensive understanding of the electronic properties of these codoped ZnS compounds.

Under the GGA approximation, the total density of states (Fig. 4c and 5c) reveals that both Zn_0.875_Mn_0.0625_Mo_0.0625_S, and Zn_0.875_Mn_0.0625_Ni_0.0625_S, systems exhibit spin-polarized electronic structures, a property that is highly desirable for spintronic applications. The partial density of states (PDOS) analysis shows that the distribution of the 3d and 4d orbitals of the codopant atoms is similar to that observed in the single-doping case, with a slight shift of the Mn-3d majority spin states toward lower energies in Zn_1−2*x*_Mn_*x*_Mo_*x*_S. Furthermore, the polarization of Mo-4d minority spin states at the Fermi level, which lies close to the bottom of the conduction band, suggests that Zn_1−2*x*_Mn_*x*_Mo_*x*_S it behaves as an n-type material. Conversely, Zn_1−2*x*_Mn_*x*_Ni_*x*_S exhibits a p-type character, as the Ni-3d orbitals are polarized near the top of the valence band.

The similarity in orbital distributions between the single-doped and codoped systems provides valuable insight into the underlying mechanism governing ferromagnetism in these materials. This observation strongly excludes the Zener double-exchange mechanism and instead supports p–d hybridization as the dominant origin of magnetic coupling in both systems. Furthermore, the oxidation state analysis, which remains consistent across both single- and codoped configurations, reinforces this conclusion, confirming that the magnetic interaction in Zn_1−2*x*_Mn_*x*_Mo_*x*_S and Zn_1−2*x*_Mn_*x*_Ni_*x*_S is mainly driven by this p–d hybridization mechanism.

Subsequently, the total and partial densities of states were recalculated using the modified Becke–Johnson (mBJ) potential to obtain a more accurate estimation of the band gap, which is crucial for a reliable description of the optical and thermoelectric properties of these compounds. The significant increase in the band gap obtained with the mBJ potential is expected, as mBJ systematically corrects the well-known band-gap underestimation of GGA. This correction is particularly effective for systems involving localized and moderately correlated 3d/4d states, such as the transition-metal dopants considered here.

**Fig. 6:**
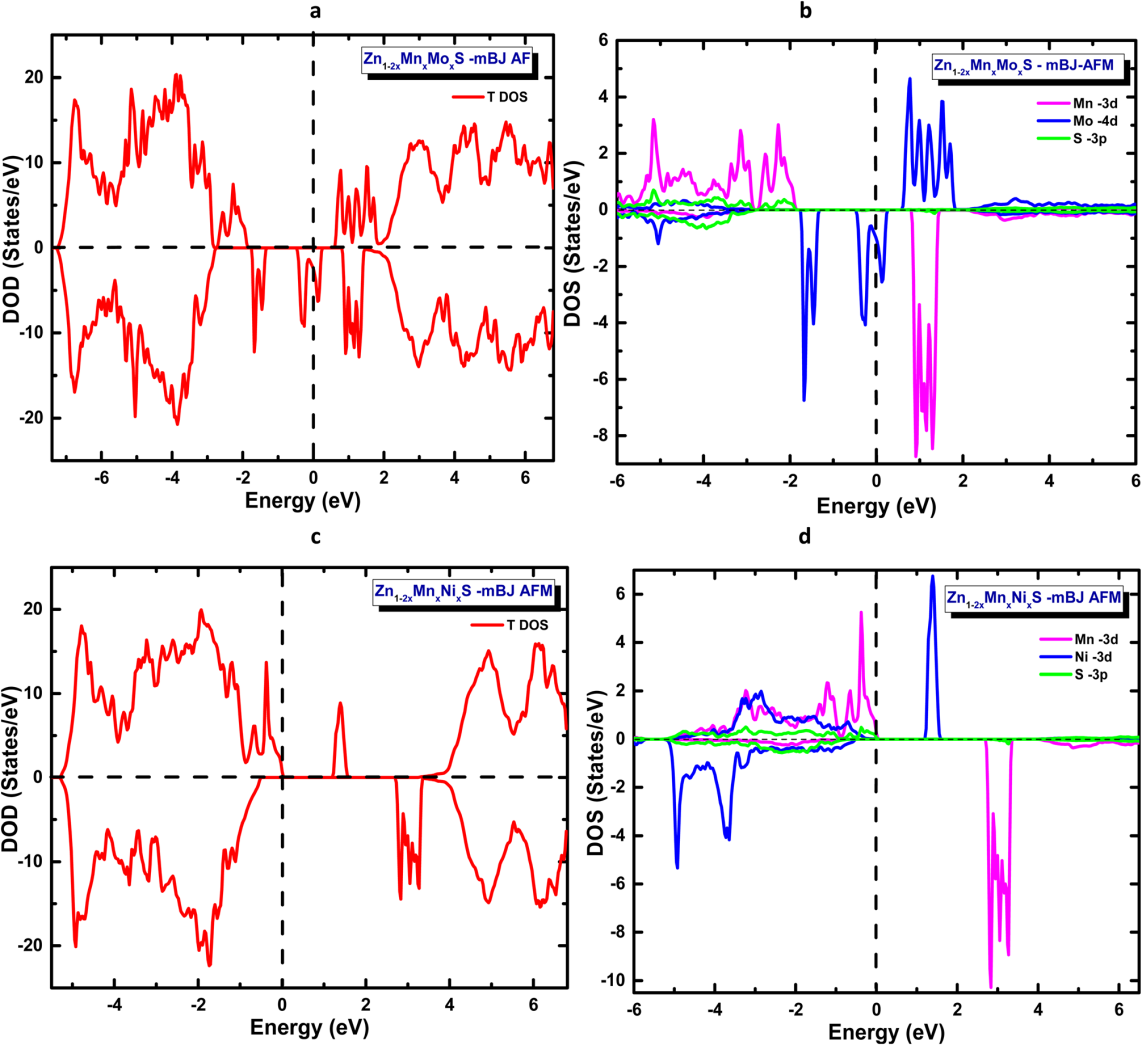
mBJ-calculated total and partial density of states in the antiferromagnetic (AFM) configuration: (a) total DOS and (b) partial DOS for ZnS codoped with Mn and Mo, and (c) total DOS and (d) partial DOS for ZnS codoped with Mn and Ni.

The mBJ-derived total and partial densities of states for Zn_1−2*x*_Mn_*x*_Mo_*x*_S (Fig. 6a and b) confirm that the system retains its half-metallic character, exhibiting an orbital distribution similar to that obtained from the GGA calculations. The main difference lies in the band gap value: while the GGA approach yields a band gap of approximately 1.6 eV, the mBJ correction increases it to about 2.6 eV. Despite this variation, the system maintains its n-type conduction character, in agreement with the previous findings.

In contrast, the mBJ-calculated density of states for Zn_1−2*x*_Mn_*x*_Ni_*x*_S (Fig. 6c) reveals that the system loses its half-metallic character owing to the pronounced splitting between the majority and minority Ni-3d spin states, which suppresses the spin polarization at the Fermi level. Nevertheless, Zn_1−2*x*_Mn_*x*_Ni_*x*_S retains its p-type conduction behaviour, with an estimated band gap of about 3.0 eV. These results demonstrate that the electronic structure and magnetic behaviour of Zn_1−2*x*_Mn_*x*_A_*x*_S (A = Mo, Ni) are highly sensitive to the nature of the codopant atoms, highlighting the crucial role of codoping in tailoring multifunctional materials for spintronic applications.

**Fig. 7:**
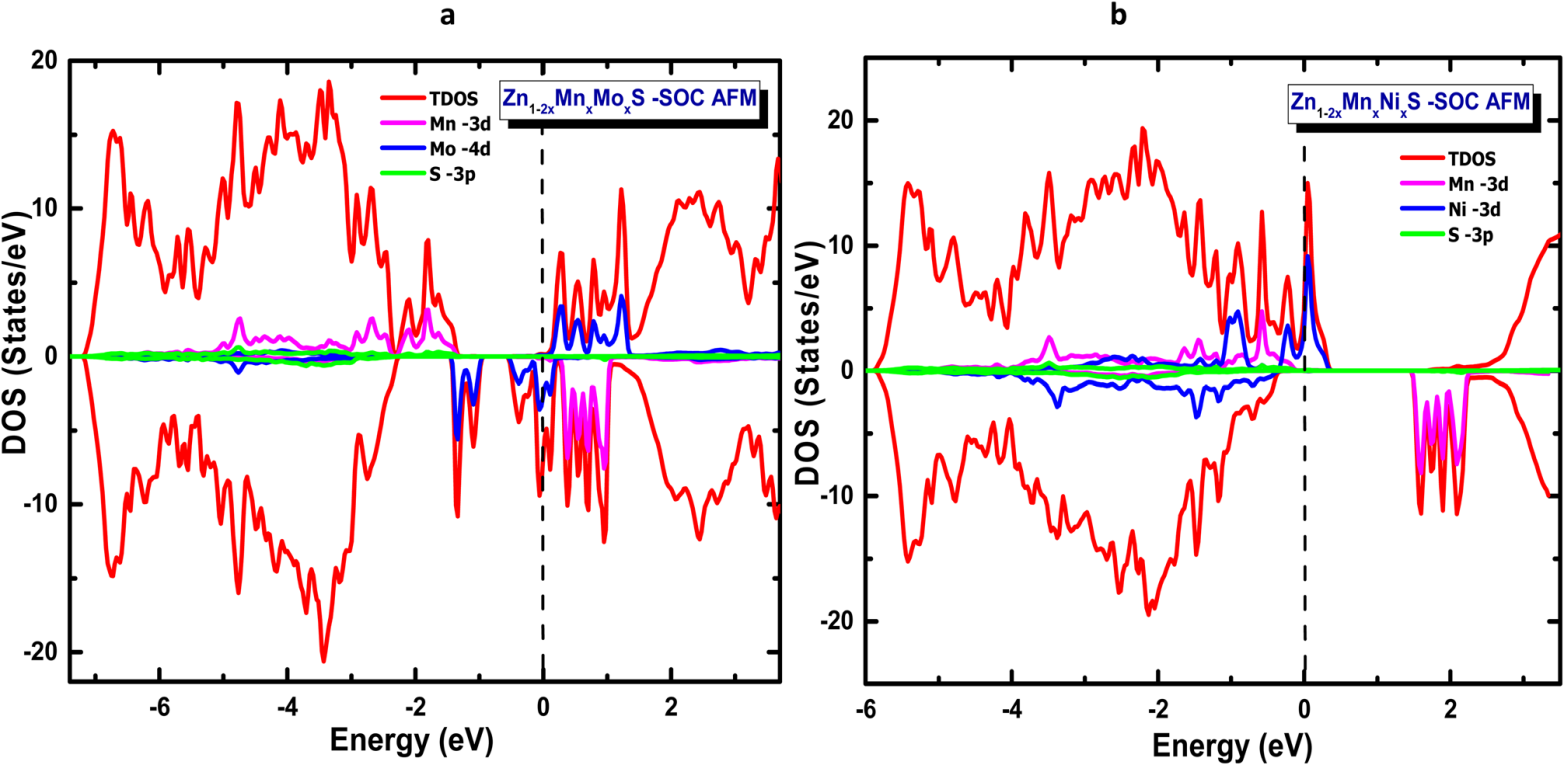
Spin–orbit–coupling (SOC)-calculated total and partial density of states for ZnS codoped with (a) Mn–Mo and (b) Mn–Ni in the AFM configuration.

The effect of spin–orbit coupling on the electronic structure of Mn–Mo- and Mn–Ni-codoped ZnS was investigated within the GGA framework. The calculated total and partial density of states in the AFM configuration indicate that the inclusion of SOC does not induce any qualitative modification of the electronic character of the systems. As illustrated in Fig. 7, the states in the vicinity of the Fermi level remain predominantly governed by Mn 3d orbitals, with additional contributions from Mo 4d or Ni 3d states depending on the codopant.

The primary impact of SOC is limited to a slight splitting and redistribution of the d states near the Fermi energy, while the overall band topology is preserved. Importantly, the inclusion of SOC preserves the half-metallic character and the spin polarization of the systems, further confirming the robustness of their electronic structure. The magnetic moments obtained at the GGA + SOC level are summarized in Table 5, showing only marginal variations with respect to the GGA values.

**Table 5:** Calculated total and atom-resolved magnetic moments for Mn–Mo- and Mn–Ni-codoped ZnS in the AFM configuration, including spin–orbit coupling (GGA + SOC)

Property	Mn/Mo (SOC)	Mn/Ni (SOC)
Total moment *M*_tot_ (µB)	1.040	3.005
Local *M*_Mn_ (µB)	4.176	4.188
Local *M*_Mo_ (µB)	−2.299	_
Local *M*_Ni_ (µB)	_	−1.287

Overall, the combined GGA, mBJ, and GGA + SOC results demonstrate the robustness of the magnetic and electronic properties of Mn–Mo- and Mn–Ni-codoped ZnS, highlighting the stability of their spin polarization and tunable electronic character, which are essential for potential spintronic applications.

### 3.2 Optical properties

**Fig. 8:**
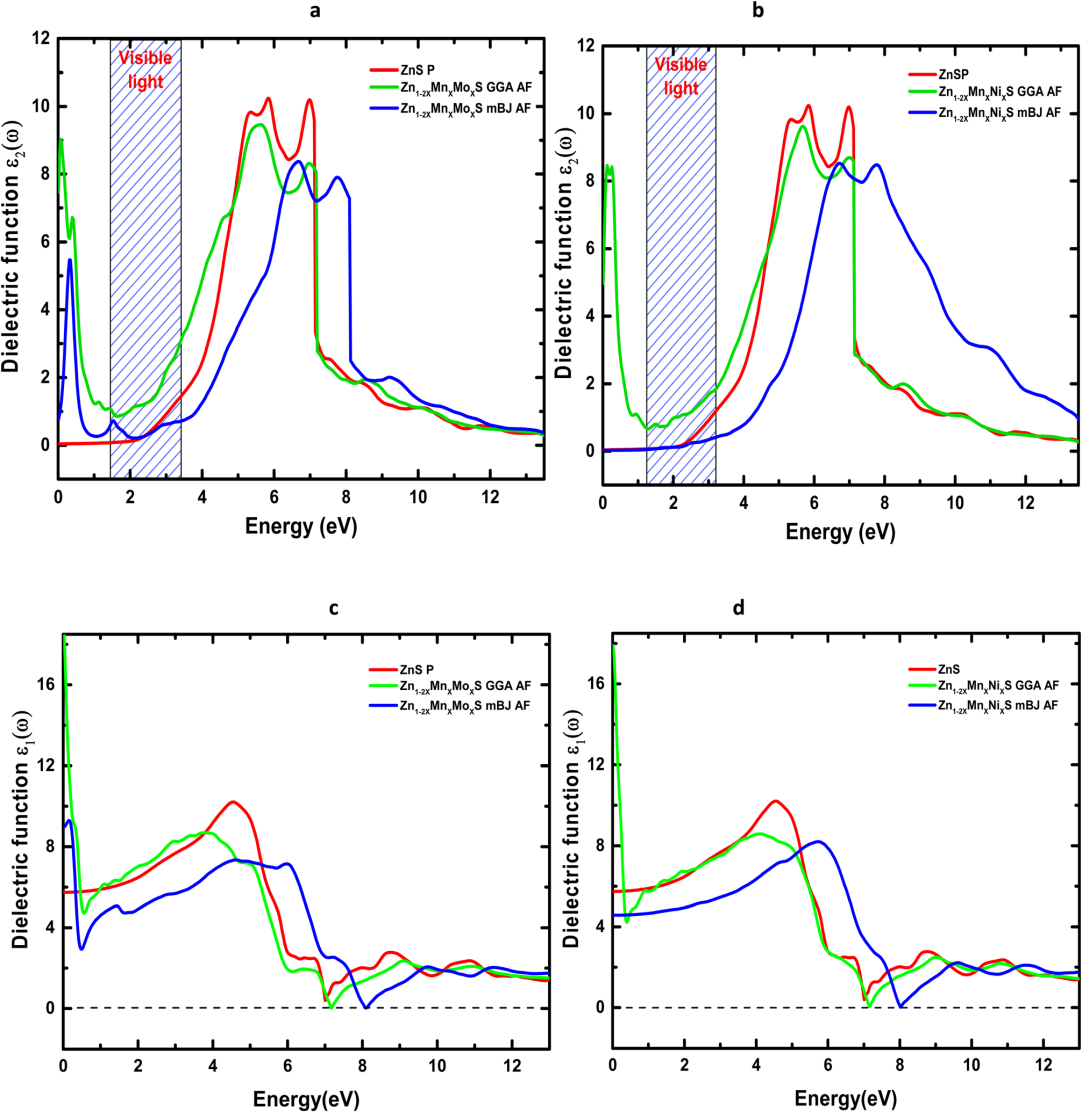
Real and imaginary parts of the dielectric function *ε*(*ω*) calculated using GGA and mBJ for: (a) *ε*_1_(*ω*) of Zn_1−2*x*_Mn_*x*_Mo_*x*_S, (b) *ε*_1_(*ω*) of Zn_1−2*x*_Mn_*x*_Ni_*x*_S, (c) *ε*_2_(*ω*) of Zn_1−2*x*_Mn_*x*_Mo_*x*_S and (d) *ε*_2_(*ω*) of Zn_1−2*x*_Mn_*x*_Ni_*x*_S in the AFM configuration.

In this section, the optical response of the antiferromagnetic half-metallic systems is analysed on the basis of spin-polarized calculations performed using the GGA-PBE and TB-mBJ approaches. The frequency-dependent behaviour was obtained from the complex dielectric function *ε*(*ω*) = *ε*_1_(*ω*) + i*ε*_2_(*ω*), computed within the linear-response formalism.^41,60,61^ Owing to the intrinsic anisotropy of the wurtzite structure *ε*_*xx*_ = *ε*_*yy*_ ≠ *ε*_*zz*_, the optical spectra discussed below correspond to the averaged dielectric component, defined as *ε*(*ω*) = [*ε*_*xx*_(*ω*) + *ε*_*yy*_(*ω*) + *ε*_*zz*_(*ω*)/3. The imaginary part *ε*_2_(*ω*) accounts for interband transitions and is therefore highly sensitive to the electronic modifications induced by Mo and Ni codoping. For reference, the *ε*_2_(*ω*) spectrum of pristine ZnS is shown in Fig. 8a and b. The real part *ε*_1_(*ω*) describes the dispersion behaviour, static dielectric response, and low-energy polarizability, while the remaining optical constants (refractive index, absorption coefficient, reflectivity, and energy-loss function) were derived from *ε*(*ω*) using standard relations.^62,63^

To provide a consistent comparison between pure and codoped systems, both *ε*_1_(*ω*) and *ε*_2_(*ω*) were examined over the photon-energy interval 0–12 eV. Pristine ZnS displays several distinct features in *ε*_2_(*ω*), originating from interband transitions between occupied and unoccupied states. The intense structure around 5.5 eV is associated with electronic excitations from S-p states in the valence band to Zn-d states in the conduction band, while the peak near 6.7 eV corresponds to higher-energy p–d transitions, in agreement with previous studies.^64^ Upon introducing (Mn,Mo) codoping, both GGA and TB-mBJ calculations reveal the emergence of an additional low-energy peak that reflects the half-metallic nature of the system. A similar tendency is observed for the (Mn,Ni)-codoped configuration, although the low-energy contribution is more pronounced within GGA-PBE. In both codoped materials, the *ε*_2_(*ω*) intensity decreases steadily as photon energy increases, indicating reduced optical activity at higher energies and suggesting enhanced absorption of infrared and low-energy visible photons compared with pristine ZnS. The real part *ε*_1_(*ω*) further highlights the impact of transition-metal codoping. Pure ZnS exhibits a static dielectric constant of 5.74, whereas Mn–Mo codoping enhances this value to 18.91 within GGA-PBE and to 8.97 using TB-mBJ, with a pronounced peak near 3.9 eV. For the (Mn, Ni)-codoped system, GGA predicts a high static value of 17.91, while TB-mBJ yields a lower value of 4.58; both methods produce a characteristic feature around 4 eV.

These trends confirm the well-established inverse relationship between the static dielectric constant and the electronic band gap: decreasing the band gap enhances polarizability and increases *ε*_2_(0), whereas widening the gap lowers the low-energy dielectric response. This behaviour agrees with the electronic structures previously obtained for the Zn_1−2*x*_Mn_*x*_Mo_*x*_S and Zn_1−2*x*_Mn_*x*_Ni_*x*_S systems. Overall, codoping substantially modifies the dielectric dispersion of ZnS, shifting the absorption edge and altering the spectral profile of *ε*_1_(*ω*).

**Fig. 9:**
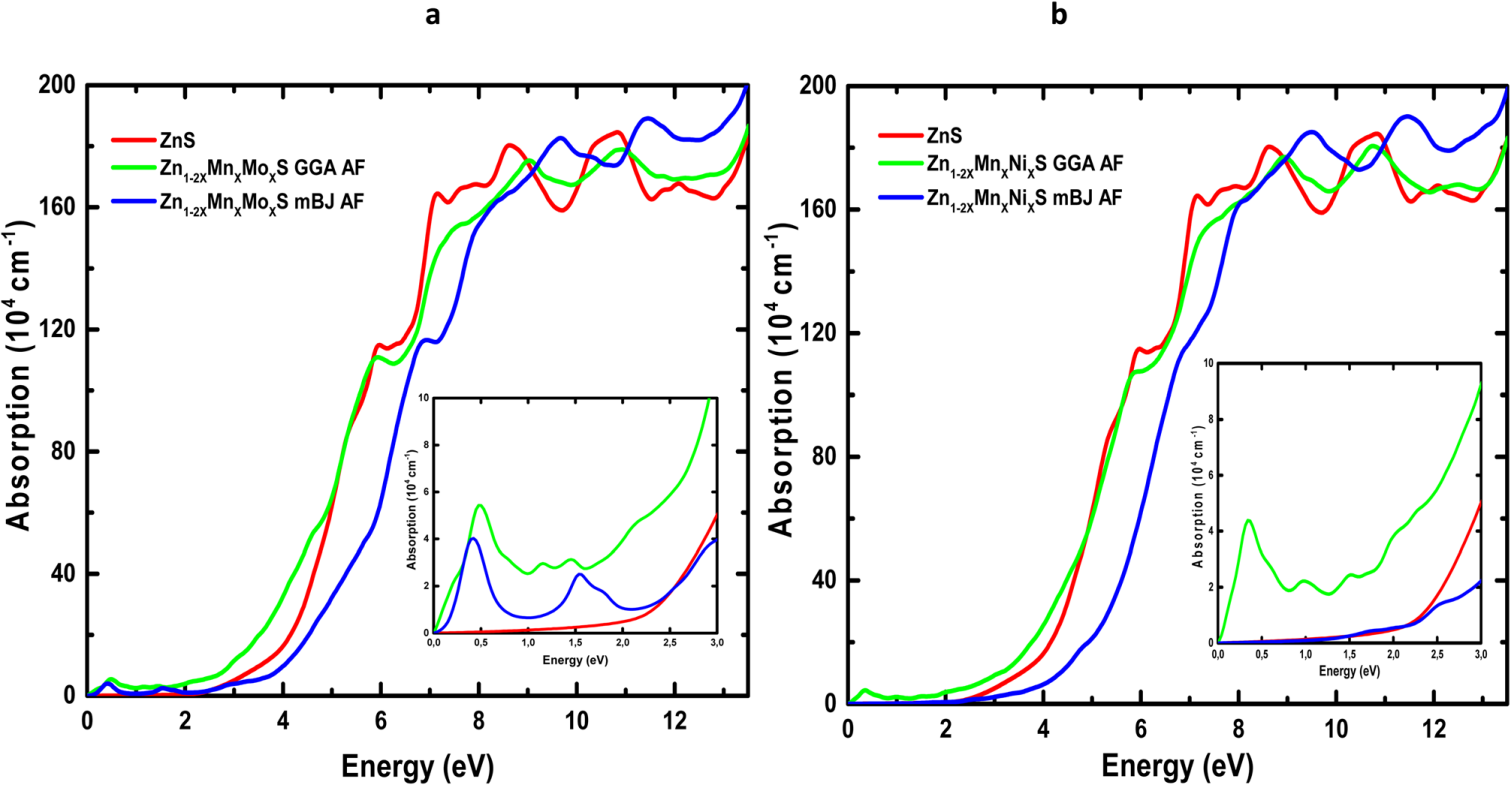
Optical absorption spectra of Codoped ZnS: (a) Zn_1−2*x*_Mn_*x*_Mo_*x*_S and (b) Zn_1−2*x*_Mn_*x*_Ni_*x*_S (*x* = 0.0625).

Understanding the real and imaginary parts of the dielectric function enables the determination of key optical parameters, including the absorption coefficient *α*(*ω*), as shown in Fig. 9. The optical absorption spectra of pure, (Mn,Mo)-codoped, and (Mn,Ni)-codoped ZnS were calculated using both approximations. Pure ZnS exhibits a broad direct band gap, reflected by an absorption edge near 3 eV, and shows very weak absorption in the visible region, which limits its applicability in visible-light-driven devices. Its strong ultraviolet absorption, consistent with previous reports,^65,66^ confirms its suitability for UV optoelectronic technologies.

Codoping ZnS with (Mn,Mo) or (Mn,Ni) results in a marked enhancement of the maximum absorption intensity, accompanied by a slight shift toward higher photon energies. Additional absorption features appear in the infrared region (0–1.45 eV), arising from d–d electronic transitions of the dopant elements, as supported by the PDOS analysis. The codoped systems therefore display broadband absorption spanning the infrared to UV range, which significantly expands their applicability to optoelectronic devices operating across multiple spectral windows.

The incorporation of transition-metal dopants induces a visible redshift, indicating modified electronic transitions toward lower photon energies. Moreover, the high absorption coefficient in the UV range highlights the strong photon-to-electron conversion potential of these materials, making them promising candidates for high-efficiency photonic and photovoltaic applications. Finally, the distinct infrared absorption peaks suggest that (Mn,Mo)- and (Mn,Ni)-codoped ZnS may also be effective for infrared sensing or energy-harvesting technologies, further broadening their functional versatility.

**Fig. 10:**
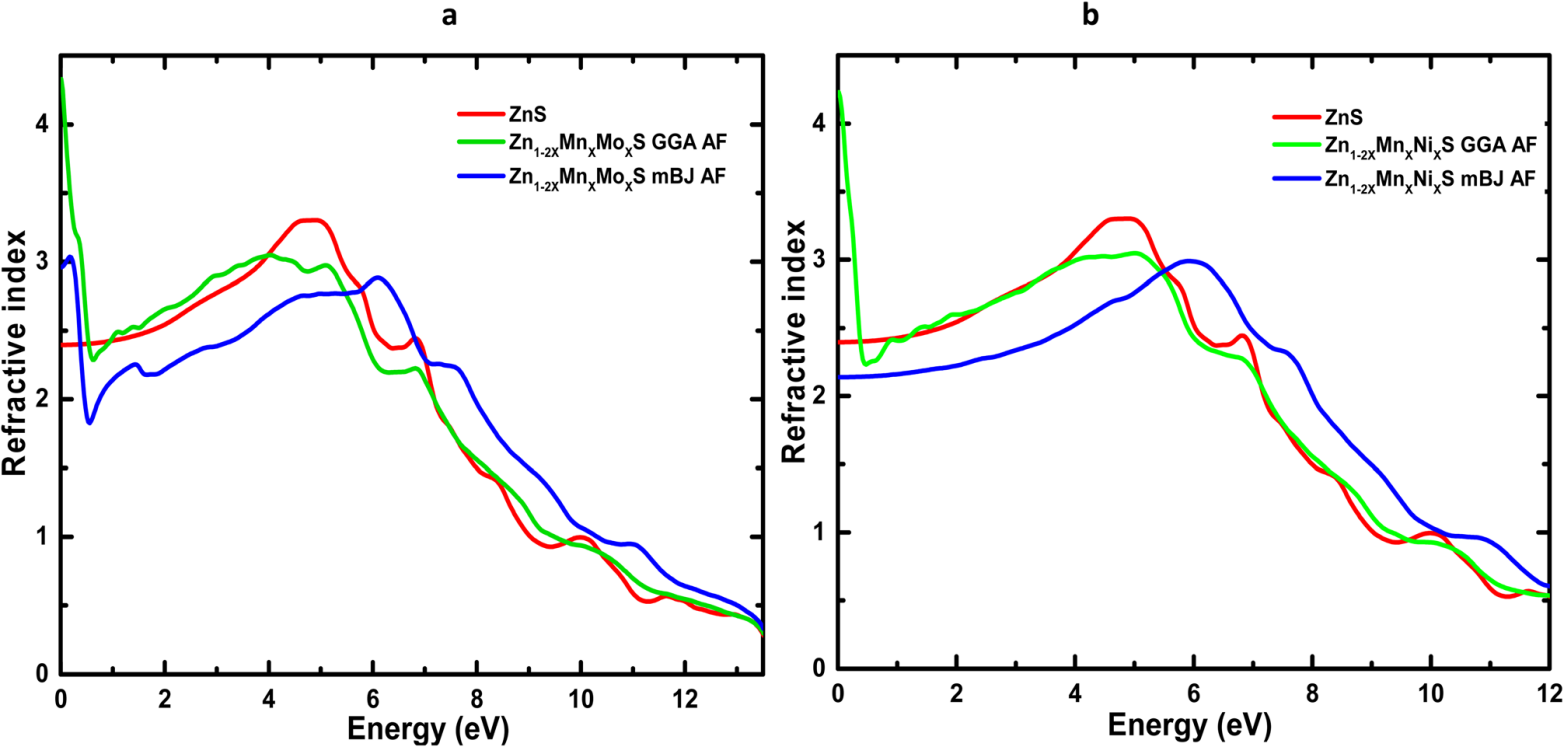
Refractive index spectra of codoped ZnS: (a) Zn_1−2*x*_Mn_*x*_Mo_*x*_S and (b) Zn_1−2*x*_Mn_*x*_Ni_*x*_S (*x* = 0.0625).

**Fig. 11:**
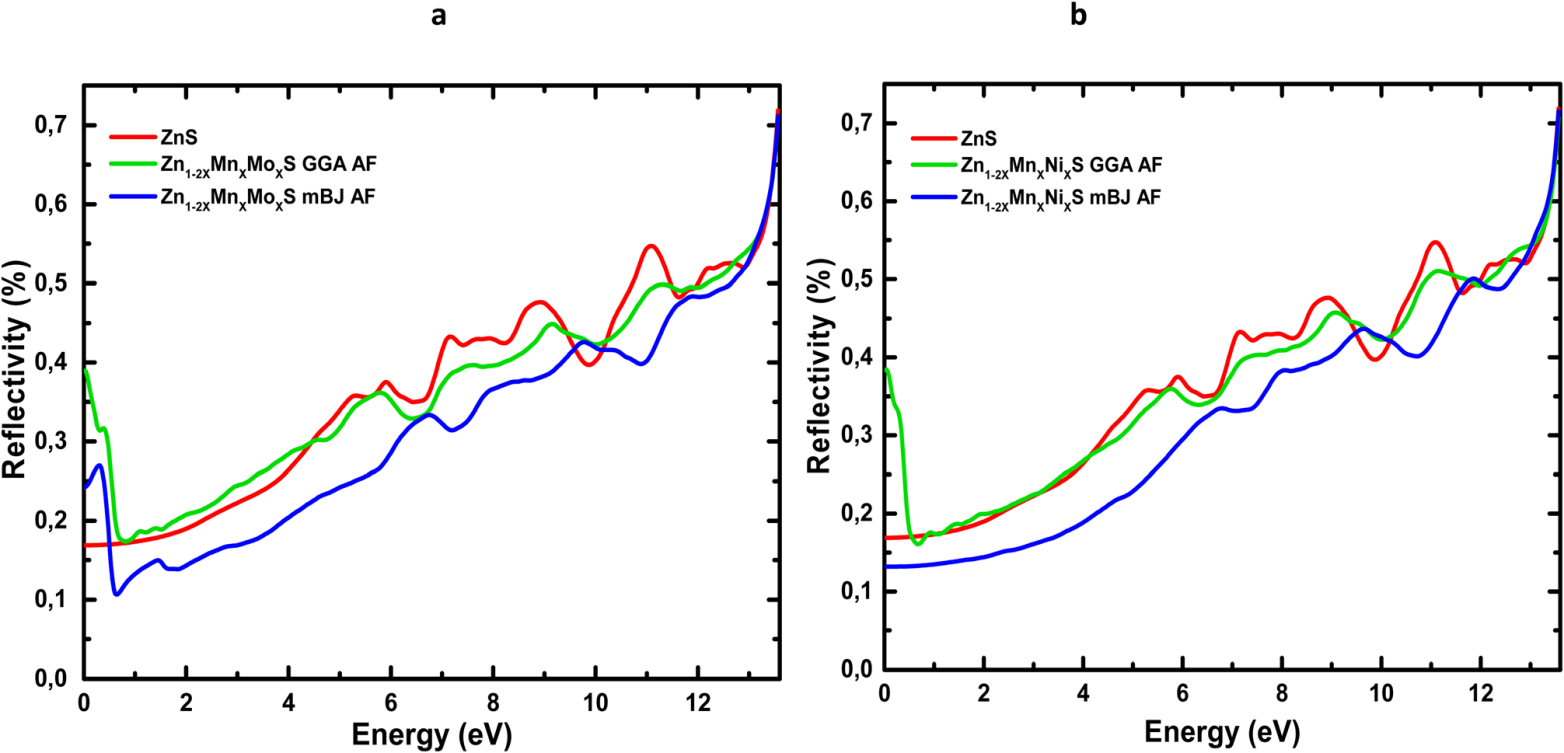
Reflectivity spectra of codoped ZnS: (a) Zn_1−2*x*_Mn_*x*_Mo_*x*_S and (b) Zn_1−2*x*_Mn_*x*_Ni_*x*_S (*x* = 0.0625).

Table .6 summarizes the principal optical parameters, including the absorption coefficient (*α*), refractive index (*n*), energy-loss function (*L*), and the real (*ε*_1_) and imaginary (*ε*_2_) parts of the dielectric function, calculated for pure ZnS and its (Mn,Mo)- and (Mn,Ni)-codoped derivatives at photon energies of 0, 3, and 6 eV using the GGA and TB-mBJ exchange–correlation schemes. The variation of the refractive index *n*(*ω*) and reflectivity *R*(*ω*) as a function of photon energy for ZnS, Zn_1−2*x*_Mn_*x*_Mo_*x*_S, and Zn_1−2*x*_Mn_*x*_Ni_*x*_S (*x* = 0.0625) is presented in Fig. 10 and 11.

**Table 6:** Calculated optical properties of ZnS and co-doped Zn_1−2*x*_Mn_*x*_A_*x*_S (A = Mo, Ni) using GGA and mBJ. *ε*_1_ and *ε*_2_ are the real and imaginary parts of the dielectric function, *α* is the absorption coefficient, *n* is the refractive index, and *R* is the reflectivity

Energy (eV)	Compound	*ε*_1_ (GGA)	*ε*_1_ (mBJ)	*ε*_2_ (GGA)	*ε*_2_ (mBJ)	*α* (GGA)	*α* (mBJ)	*n* (GGA)	*n* (mBJ)	*R* (%) (GGA)	*R* (%) (mBJ)
0	ZnS	5.74	—	0	—	0	—	2.40	—	16	—
	Zn_1−2*x*_Mn_*x*_Mo_*x*_S	18.91	8.97	7.47	0.77	0.10	0.02	4.33	2.96	39	24
	Zn_1−2*x*_Mn_*x*_Ni_*x*_S	17.91	4.58	4.97	0.03	0.08	0.00	4.23	2.14	38	13
3	ZnS	7.68	—	0.93	—	5.08	—	2.78	—	22	—
	Zn_1−2*x*_Mn_*x*_Mo_*x*_S	8.27	5.69	2.21	0.63	11.61	3.98	2.90	2.39	24.5	17
	Zn_1−2*x*_Mn_*x*_Ni_*x*_S	7.52	5.46	1.69	0.34	9.31	2.25	2.76	2.34	23	16
6	ZnS	2.79	—	9.49	—	114.76	—	2.53	—	37	—
	Zn_1−2*x*_Mn_*x*_Mo_*x*_S	1.94	7.14	8.33	6.01	110.60	63.66	2.29	2.87	35	28.5
	Zn_1−2*x*_Mn_*x*_Ni_*x*_S	2.78	7.88	8.59	6.11	107.62	62.14	2.43	2.99	34	29

**Fig. 12:**
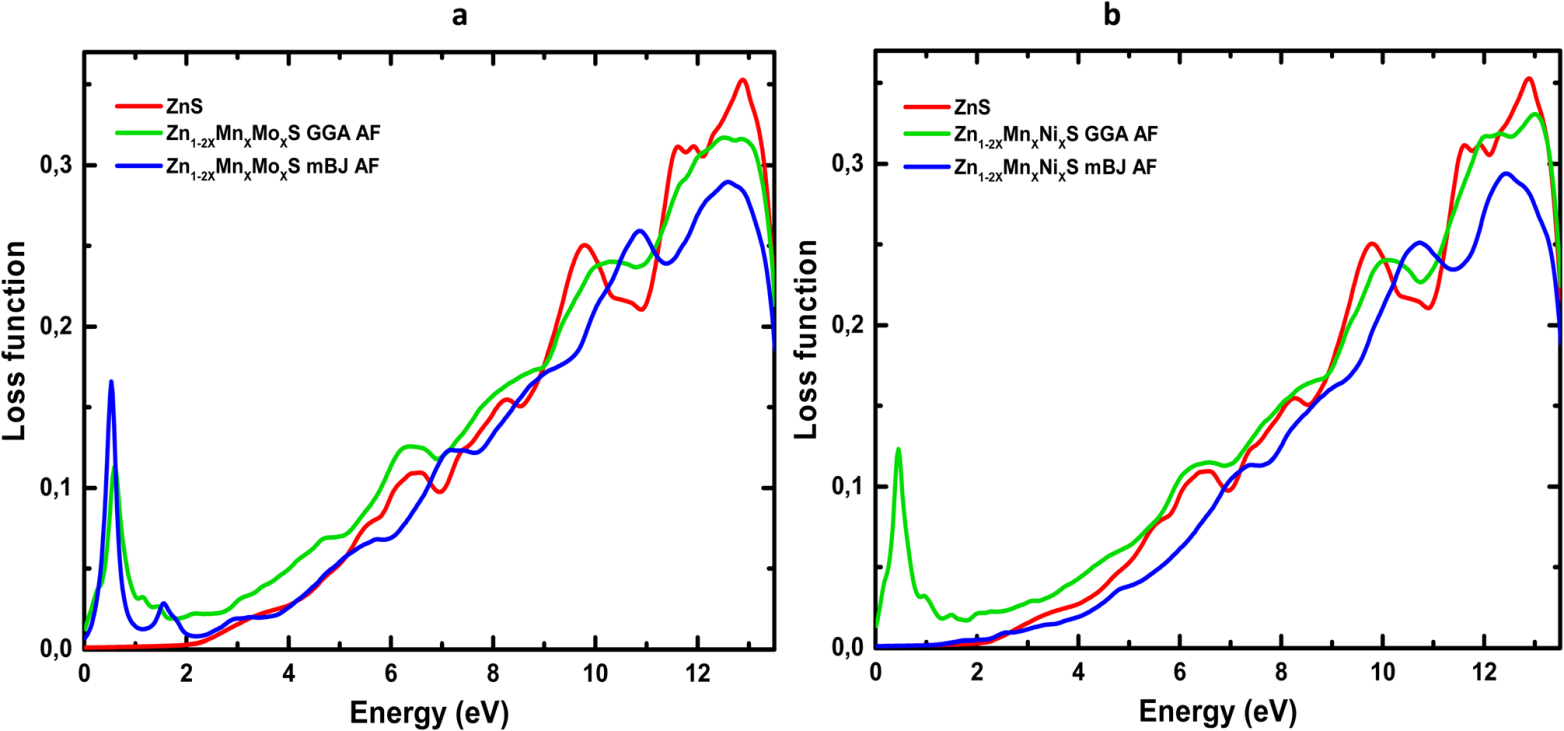
Energy loss function spectra of codoped ZnS: (a) Zn_1−2*x*_Mn_*x*_Mo_*x*_S and (b) Zn_1−2*x*_Mn_*x*_Ni_*x*_S (*x* = 0.0625).

The evolution of *n*(*ω*) (Fig. 10) shows that codoping with (Mn,Mo) and (Mn,Ni) induces a marked enhancement of the static refractive index, consistent with the values listed in Table 6. This increase reflects the higher electronic polarizability and light transmission arising from the dopant-induced localized states associated with transition-metal ions. In addition, the TB-mBJ functional, through its improved description of the band gap, shifts the characteristic *n*(*ω*) peaks to higher energies, confirming the strong dependence of calculated optical responses on the choice of exchange–correlation functional. Reflectivity, which quantifies the fraction of incident light reflected by the material, also exhibits a substantial modification upon codoping (Fig. 11). In the visible range, the reflectance of Zn_1−2*x*_Mn_*x*_Mo_*x*_S reaches 39% within GGA and 24% within TB-mBJ, compared to 16% for pristine ZnS. A comparable enhancement is observed for Mn–Ni codoping, where *R* increases up to 38% under GGA. These increases may be attributed to antiferromagnetic interactions between Mn ions in the ZnS host and the Mo/Ni dopants, which modify the electronic structure near the Fermi level. At higher photon energies (6–12 eV), the reflectivity rises sharply for all systems, mainly due to interband electronic transitions, in agreement with the negligible values of *ε*_1_ and *ε*_2_ in this energy window. The energy loss function, *L*(*ω*), measures the probability that an incoming electron transfers energy to the system through plasmon excitations or interband transitions.^67^ As shown in Fig. 12, *L*(*ω*) increases progressively with photon energy, indicating successive electronic excitations. Codoping introduces additional peaks and amplifies *L*(*ω*) in both the visible and infrared regions, reflecting the appearance of new electronic states within the band structure and confirming the enhancement of optical and plasmonic activity.

Overall, these results demonstrate that codoping substantially modifies the optical response of ZnS, enhancing its refractive, reflective and plasmonic characteristics, which are highly desirable for optoelectronic applications such as solar cells, optical sensors and reflective or photonic devices.

### 3.3 Thermoelectric properties

Thermoelectric materials are receiving considerable attention in the current context of the energy transition owing to their capacity to directly convert thermal energy into electrical power without moving parts or pollutant emissions. Because of these advantages, they are promising candidates for waste-heat harvesting and for the development of autonomous energy devices.^68–70^ In this work, the BoltzTraP package interfaced with the Wien2k code was employed to calculate the thermoelectric properties within the framework of semiclassical Boltzmann transport theory.^71,72^ All transport quantities were obtained using the constant relaxation time approximation with *τ* = 10^−14^ s. The efficiency of a thermoelectric material is commonly expressed through the dimensionless figure of merit, 
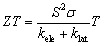
 where *S*, *σ*, *k*_ele_, *k*_lat_ and *T* denote Seebeck coefficients, electrical conductivity, electronic thermal conductivity, lattice thermal conductivity, and temperature, respectively.^32,73^

**Fig. 13:**
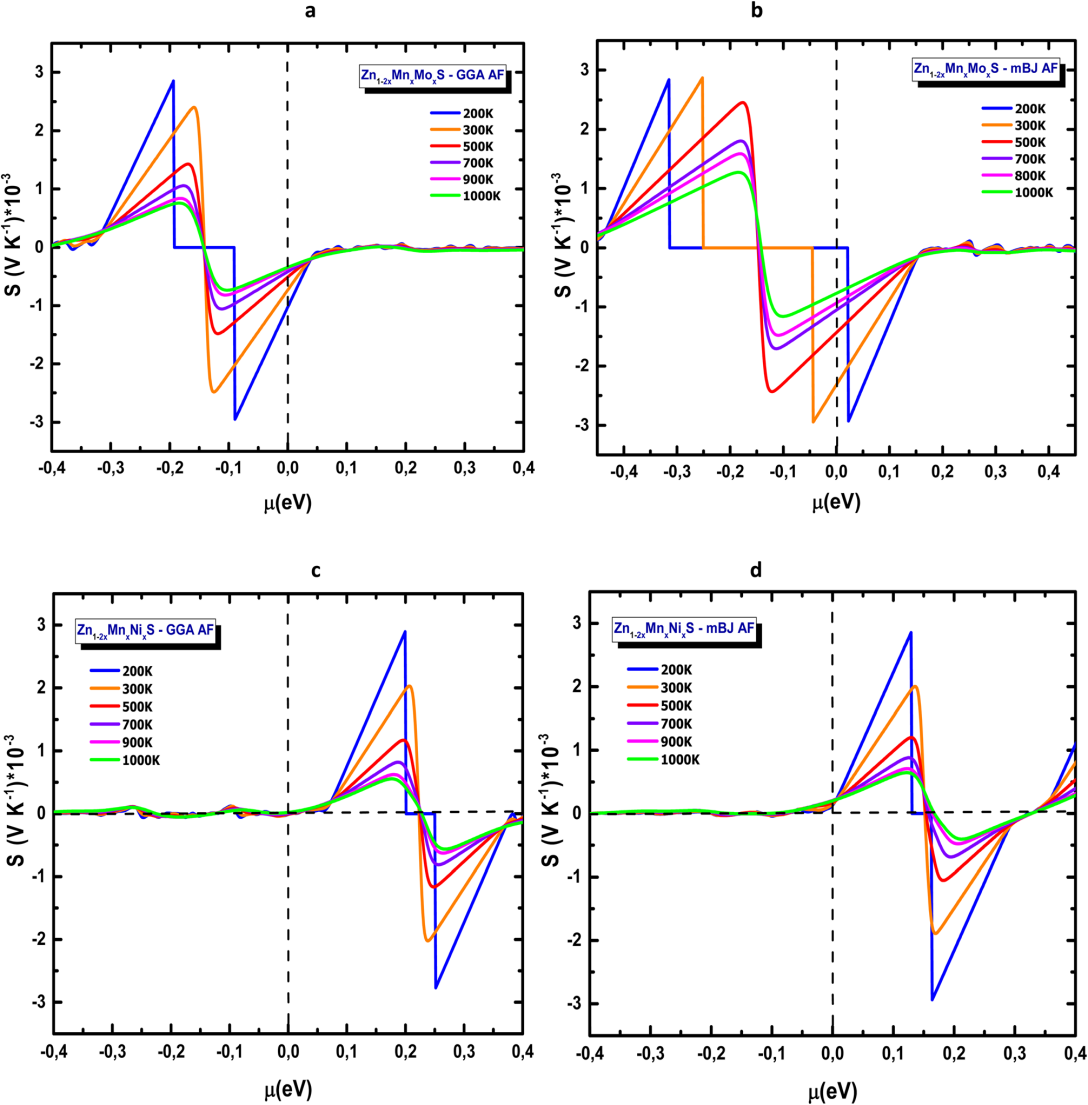
Seebeck coefficient of codoped ZnS in the AFM configuration: (a) Zn_1−2*x*_Mn_*x*_Mo_*x*_S (GGA), (b) Zn_1−2*x*_Mn_*x*_Mo_*x*_S (mBJ), (c) Zn_1−2*x*_Mn_*x*_Ni_*x*_S (GGA) and (d) Zn_1−2*x*_Mn_*x*_Ni_*x*_S (mBJ).

Fig. 13(a and b) displays the variation of the Seebeck coefficient *S* as a function of the chemical potential for the GGA-PBE and mBJ functionals. A nearly symmetric profile is observed around the Fermi level (*E* = 0, vertical dashed line), which is characteristic of half-metallic systems. At low energies (negative *µ*), *S* is positive and becomes negative near the Fermi level. Temperature has a direct influence on the amplitude and sharpness of the *S*(*µ*) curve: at 200 K, pronounced extrema are observed in the range of −3 to +3 × 10^−3^ V K^−1^, governed by strong local variations in the density of states and the concentration of carriers around *E*_f_. As temperature increases, thermal broadening smooths the S profile, reducing the intensity of the extrema while maintaining significant values up to 1000 K, which indicates potentially favourable thermoelectric performance. The mBJ functional yields a similar trend but with systematically larger amplitudes, particularly near *E* = 0, reflecting the more realistic correction of the band gap and its impact on the energy dependence of *S*. In several regions, *S* remains negative at all temperatures, pointing to hole-dominated (p-type) transport.

A comparable behaviour is obtained for the Mn–Ni codoped ZnS system (Fig. 13(c and d)). However, the strongest fluctuations of *S* appear in the positive-energy region, revealing a predominance of n-type carriers. These results confirm that both codoping configurations (Mn–Mo and Mn–Ni) preserve an enhanced thermoelectric response, particularly at low and intermediate temperatures where strong asymmetries in the carrier distribution contribute to high Seebeck values.

**Fig. 14:**
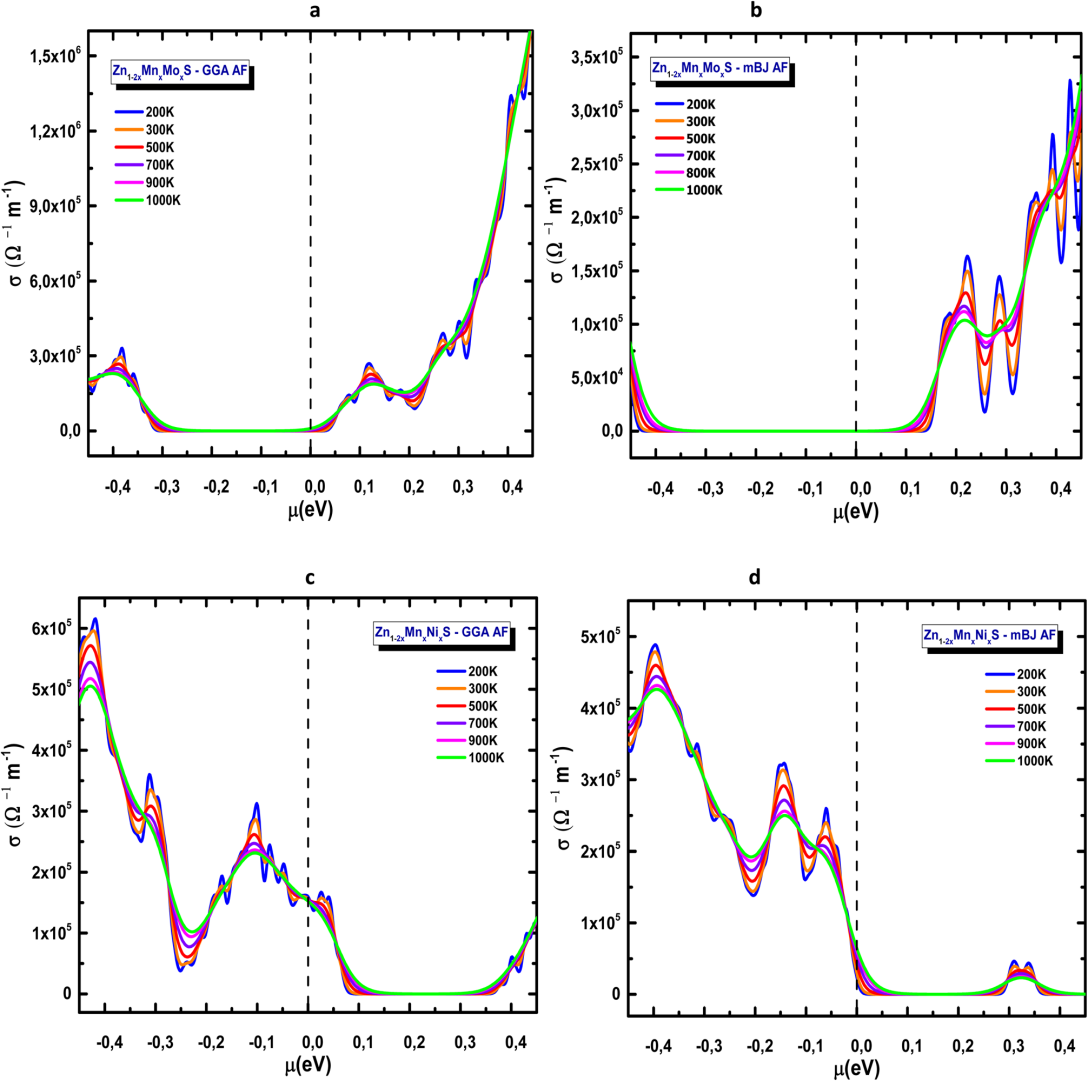
Electrical conductivity of codoped ZnS in the AFM configuration (a) Zn_1−2*x*_Mn_*x*_Mo_*x*_S (GGA), (b) Zn_1−2*x*_Mn_*x*_Mo_*x*_S (mBJ), (c) Zn_1−2*x*_Mn_*x*_Ni_*x*_S (GGA) and (d) Zn_1−2*x*_Mn_*x*_Ni_*x*_S (mBJ).

The electrical conductivity *σ* as a function of chemical potential and temperature is presented in Fig. 14. For Mn–Mo codoped ZnS (Fig. 14(a and b)), *σ* increases markedly in the conduction-band region (*E* > 0), showing pronounced peaks, whose magnitudes are significantly reduced when switching from GGA to mBJ. With GGA, the maximum conductivity exceeds 1.5 × 10^6^ m^−1^ Ω^−1^, whereas with mBJ it decreases to approximately 3 × 10^5^ m^−1^ Ω^−1^, consistent with the more accurate band-gap opening of mBJ, which reduces the carrier concentration at specific energies. Temperature influences *σ* moderately, and all curves show similar global behaviour, confirming electron-dominated transport in the conduction-band region. This observation is consistent with what the Seebeck coefficient indicates: the dominant charge carriers contributing to conduction differ between the two codoping schemes and depend on the position of *µ*. For the Zn_1−2*x*_Mn_*x*_Ni_*x*_ system (Fig. 14(c and d)), *σ* peaks in the negative-energy region, corresponding to p-type conduction, and drops sharply as *µ* approaches or exceeds 0 eV, reflecting a lower density of states in the conduction band. Conductivity increases gradually with temperature but becomes less sensitive above 700 K. As observed previously, *σ* values predicted by mBJ are systematically lower than those obtained with GGA due to the reduced carrier population resulting from a larger band gap.

**Fig. 15:**
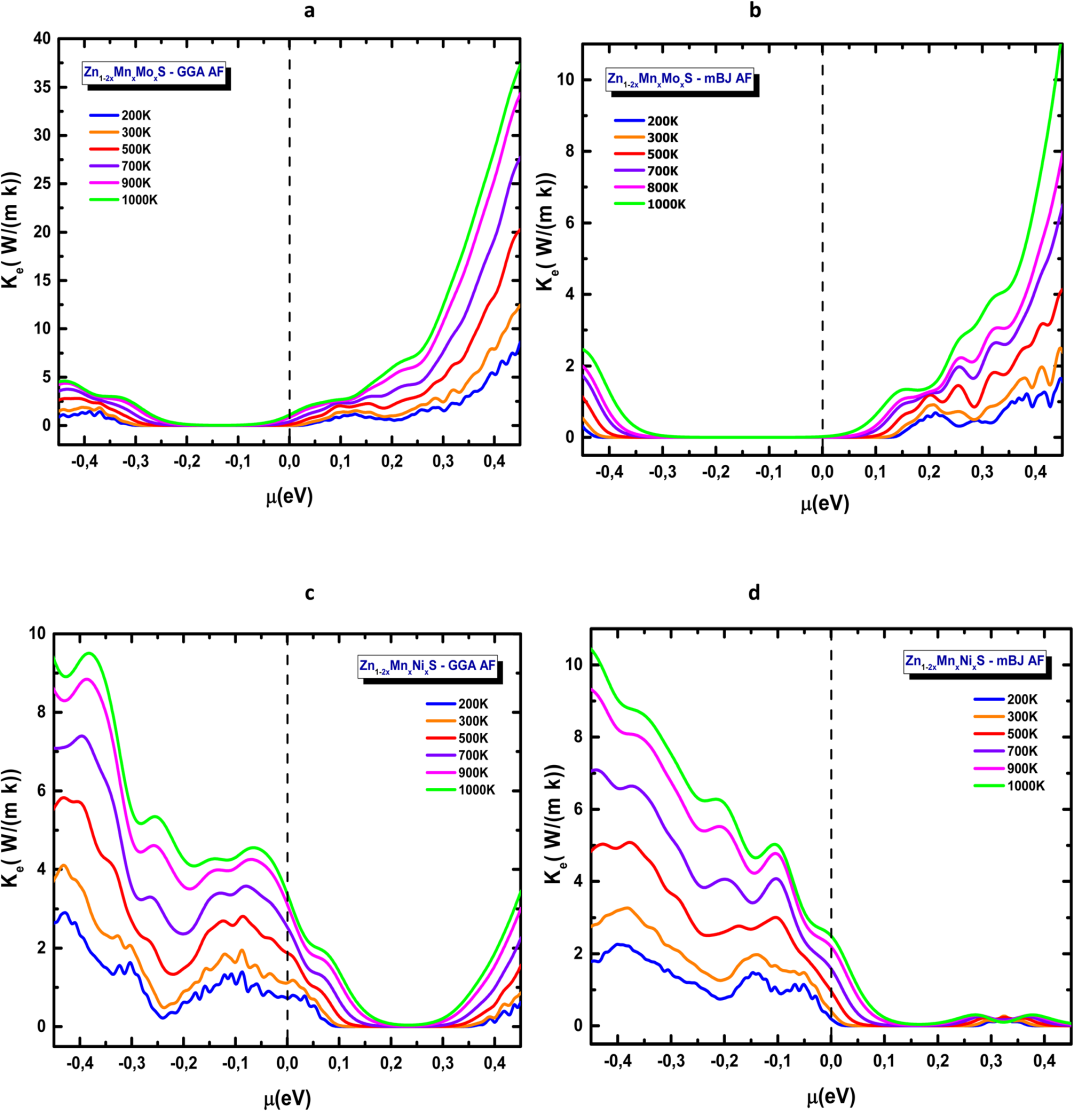
Electronic thermal conductivity of codoped ZnS in the AFM configuration: (a) Zn_1−2*x*_Mn_*x*_Mo_*x*_S (GGA), (b) Zn_1−2*x*_Mn_*x*_Mo_*x*_S (mBJ), (c) Zn_1−2*x*_Mn_*x*_Ni_*x*_S (GGA) and (d) Zn_1−2*x*_Mn_*x*_Ni_*x*_S (mBJ).

One of the key contributors to the thermoelectric figure of merit is the electronic thermal conductivity (*κ*_e_, in W m^−1^ K^−1^). Fig. 15 show the variation of *κ*_e_ with chemical potential *µ* at different temperatures for both GGA and mBJ approximations. The results confirm that electrons dominate thermal transport compared to holes: in Mn–Mo codoped ZnS, *κ*_e_ increases sharply in the conduction band, reaching its maximum. The GGA functional, which underestimates the band gap, predicts higher maxima (≈35–40 W m^−1^ K^−1^) than mBJ (≈10 W m^−1^ K^−1^ at the same energy). In contrast, in the negative chemical potential region (*µ* < 0, Fig. 15(c and d)), *κ*_e_ increases with temperature but remains relatively low at ambient conditions, reflecting predominant hole contributions and p-type behaviour. Overall, these results indicate that codoping effectively reduces *κ*_e_ compared to simple doping or pristine ZnS. This trend is consistent with previous experimental studies, such as Zhong *et al.* (2015), which reported that enhanced phonon scattering associated with the microstructure and dopant-induced defects leads to a noticeable decrease in thermal conductivity.^74,75^

**Fig. 16:**
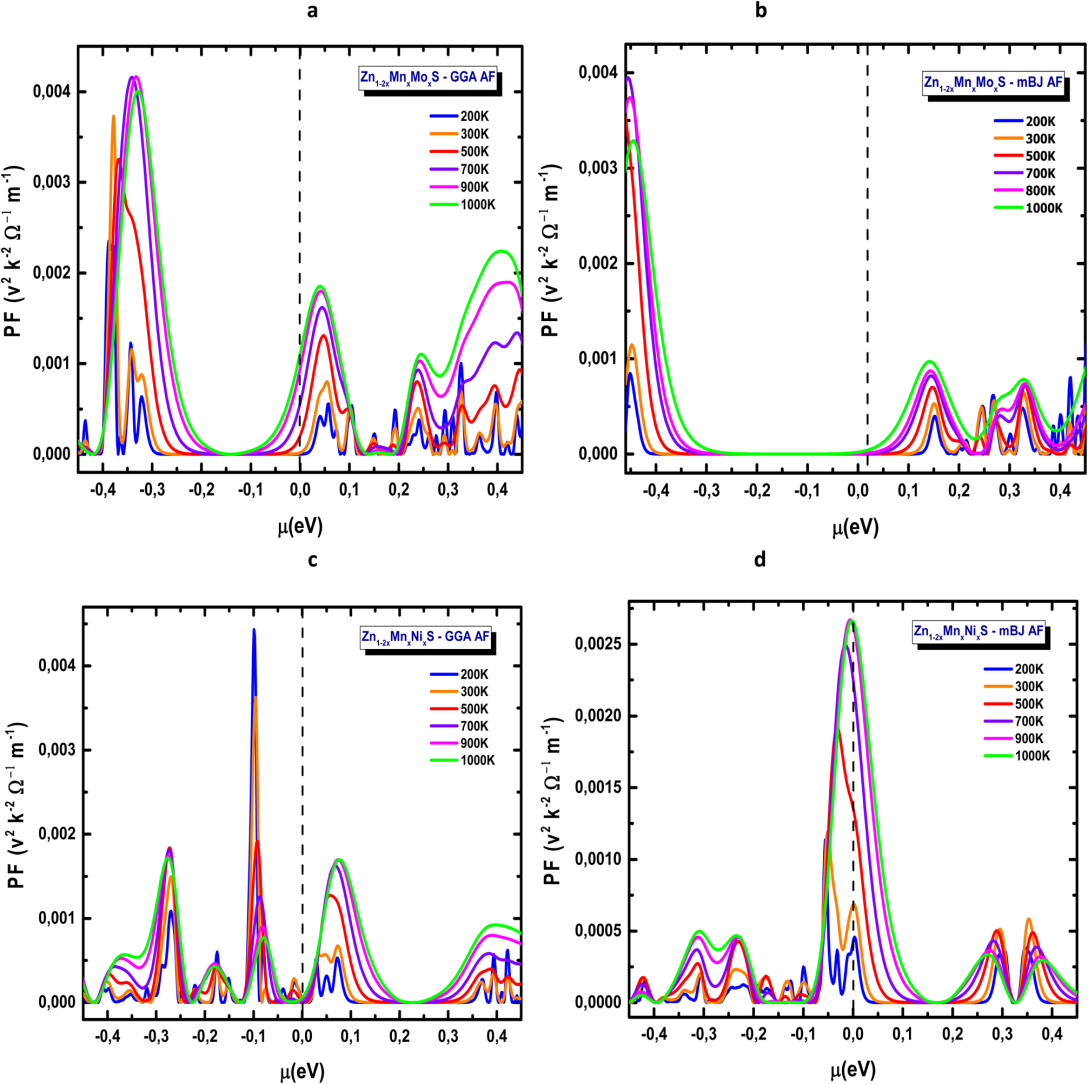
Power factor of codoped ZnS in the AFM configuration: (a) Zn_1−2*x*_Mn_*x*_Mo_*x*_S (GGA), (b) Zn_1−2*x*_Mn_*x*_Mo_*x*_S (mBJ), (c) Zn_1−2*x*_Mn_*x*_Ni_*x*_S (GGA) and (d) Zn_1−2*x*_Mn_*x*_Ni_*x*_S (mBJ).

The power factor (PF), defined as PF = *S*^2^*σ*/*τ*,^76^ is a key descriptor of thermoelectric efficiency and allows the evaluation of transport performance independently of lattice thermal losses. Fig. 16 present the variation of PF as a function of chemical potential, providing insight into the impact of Mn-based codoping on the thermoelectric response of ZnS.

For the Mn–Mo codoped system (Fig. 16(a and b)), the highest PF values occur in the negative chemical-potential region, revealing a pronounced p-type transport character. In this domain, PF reaches approximately 4 × 10^−3^ V^2^ K^−2^ Ω^−1^ m^−1^, a value that is notably high and particularly relevant for low- to mid-temperature applications. PF increases systematically with temperature, exhibiting a broad maximum between 700 and 900 K. Compared with GGA, the mBJ approximation yields sharper and broader PF peaks, reflecting its improved description of the electronic structure near the valence-band edge. In contrast, the Mn–Ni codoped compound exhibits a predominantly n-type behaviour, with the PF maximum shifting toward *µ* ≈ 0 eV, as shown in Fig. 16(c and d). Although GGA predicts slightly larger PF amplitudes, the mBJ functional provides more stable and well-defined PF profiles, offering a more realistic representation of the transport features near the conduction-band edge. This highlights the essential role of mBJ in capturing the thermoelectric characteristics of ZnS-based codoped systems with enhanced reliability.

It should be noted that the apparent differences in n-type/p-type character between the Seebeck coefficient, power factor, and DOS arise from the energy-dependent contributions of electrons and holes. Each transport property probes different aspects of carrier dynamics, so the observed trends are consistent with the electronic structure and do not indicate a contradiction.

**Fig. 17:**
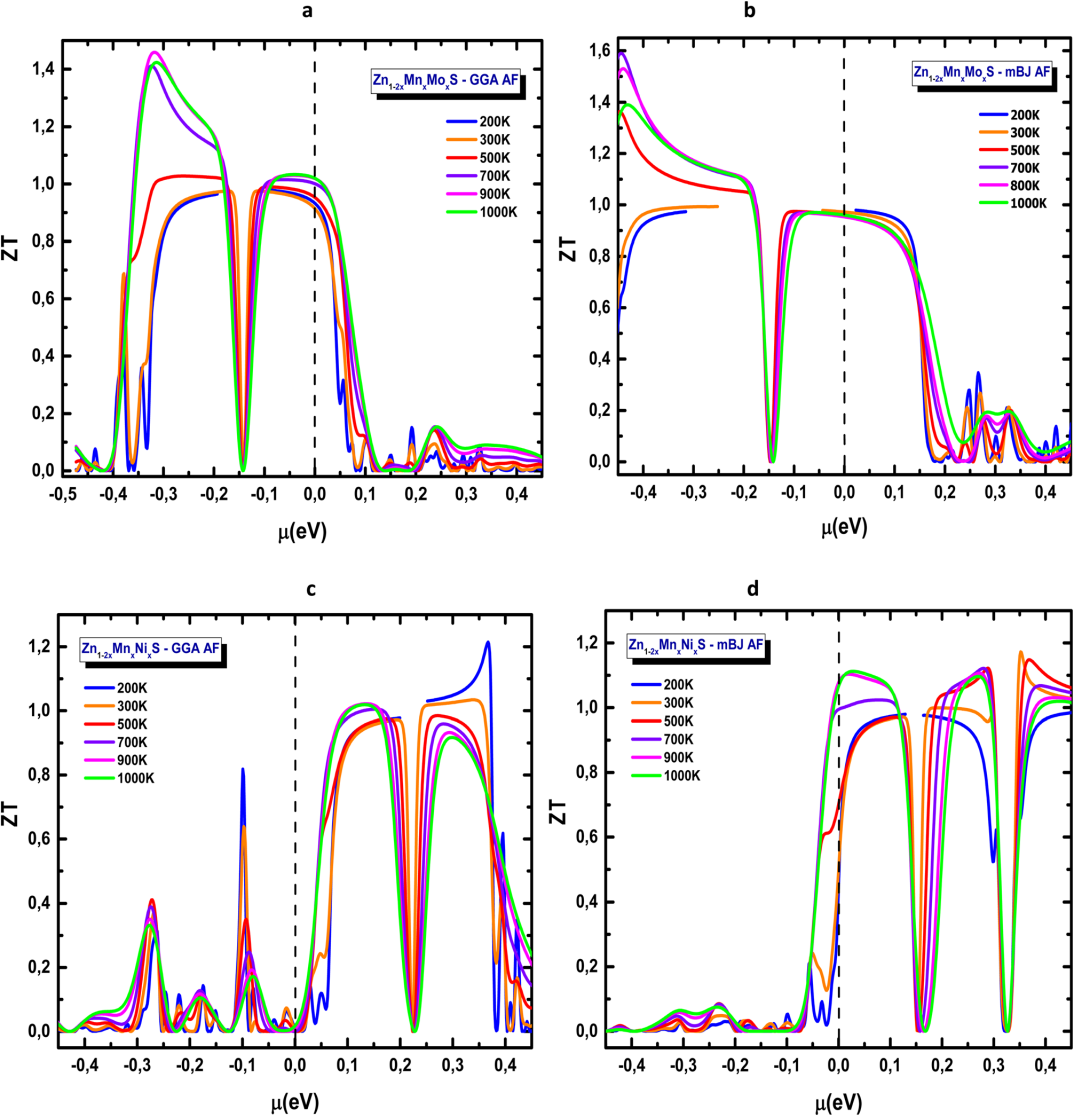
Thermoelectric figure of merit (*ZT*) of codoped ZnS in the AFM configuration: (a) Zn_1−2*x*_Mn_*x*_Mo_*x*_S (GGA), (b) Zn_1−2*x*_Mn_*x*_Mo_*x*_S (mBJ), (c) Zn_1−2*x*_Mn_*x*_Ni_*x*_S (GGA) and (d) Zn_1−2*x*_Mn_*x*_Ni_*x*_S (mBJ).

Fig. 17 present the temperature-dependent evolution of the thermoelectric figure of merit *ZT*, the most comprehensive indicator of thermoelectric performance. The curves, plotted from 200 K to 1000 K, reveal the combined effects of the Seebeck coefficient, electrical conductivity, and electronic thermal contributions. For the Mn–Mo codoped ZnS system (Fig. 17(a and b)), two distinct peaks appear around −0.3 eV and −0.1 eV within the GGA approximation, whereas mBJ yields a broader peak centred near −0.2 eV. The behaviour is clearly asymmetric between the p- and n-type regions, with the p-type domain exhibiting superior performance. The maximum *ZT* reaches approximately 1.4 (GGA) and 1.6 (mBJ), reflecting the beneficial influence of band-gap opening, particularly around the valence-band edge. In contrast, the Mn–Ni codoped ZnS compound displays a predominant n-type response for both GGA and mBJ approximations (*µ* > 0), as shown in Fig. 17(c and d). In this case, *ZT* attains similar maximum values of around 1.2 for both approximations and increases steadily with temperature. The mBJ functional produces sharper and more stable peaks, reinforcing its suitability for reliable quantitative analysis of transport properties.

Both codoping strategies markedly enhance the thermoelectric response of ZnS, confirming the efficiency of simultaneous transition-metal substitution in tailoring the electronic structure. However, the *ZT* values reported here (1.2–1.6) should be regarded as upper-limit predictions. Under the constant relaxation time approximation and without including the lattice thermal conductivity (*k*_lat_), BoltzTraP systematically overestimates *ZT*, particularly at elevated temperatures and for wide-band-gap materials such as ZnS. Therefore, the present results capture the qualitative trends with high reliability, but full phonon and *k*_lat_ calculations would be required for quantitative accuracy. These findings nonetheless indicate strong potential for applications in thermoelectric energy conversion, solid-state cooling, smart thermal sensors, and photovoltaic-integrated thermal modules.

### 3.4 Limitations and future perspectives

While the present study provides a comprehensive computational analysis, several limitations and considerations for experimental comparison must be highlighted. Although the present DFT investigation provides a coherent picture of the electronic, optical, and thermoelectric behaviour of Mn–Mo and Mn–Ni codoped ZnS, several inherent limitations should be considered. The GGA and mBJ schemes still underestimate the experimental band gap of ZnS (∼3.6–3.8 eV) owing to the approximations in the exchange–correlation treatment. While mBJ improves the band-edge description, excitonic and many-body effects-important for the onset and shape of optical absorption not included in the present calculations. Consequently, a fully quantitative agreement with experimental optical spectra may be limited. Similar underestimations of optical transitions using GGA or mBJ approaches have been widely reported in the literature, and benchmark studies based on the GW approximation (GW) and the Bethe–Salpeter equation (BSE) have demonstrated the necessity of incorporating many-body corrections to achieve a reliable description of optical spectra.^77,78^

Regarding thermoelectric transport, the *ZT* values reported here correspond to upper estimates. They rely on the constant relaxation-time approximation and exclude the lattice thermal conductivity *k*_lat_, which typically lowers the experimental *ZT*, especially in wide-gap semiconductors such as ZnS. Real samples also exhibit defect scattering, grain-boundary effects and phonon–phonon interactions that are not captured within BoltzTraP, further reducing measurable performance.

More advanced theoretical treatments, including GW- or BSE-level corrections for electronic and optical properties, and full phonon and *k*_lat_ evaluations for transport coefficients, would provide more quantitative accuracy. Experimental validation through optical absorption, Hall measurements, and thermoelectric characterization of Mn–Mo and Mn–Ni codoped ZnS would be highly valuable to confirm the trends identified in this work and to guide the design of optimized codoping strategies.

## 4. Conclusion

In summary, this study demonstrates the successful engineering of wurtzite ZnS through (Mn, Mo) and (Mn, Ni) codoping using first-principles calculations within the GGA and mBJ frameworks. Both systems exhibit half-metallic behavior under FM and AFM configurations, with the AFM phase being the most stable. The mBJ approach provides an improved band gap estimation and a more accurate depiction of the electronic structure. Codoping effectively tunes the carrier type, leading to n-type conductivity for (Mn, Mo) and p-type for (Mn, Ni). Enhanced optical absorption, reduced reflectivity, and a distinct redshift indicate improved optical activity, while the thermoelectric analysis predicts higher *ZT* values and better conversion efficiency. These results highlight the potential of codoped ZnS as an engineered multifunctional material for advanced optoelectronic, spintronic, and energy-conversion applications.

## Conflicts of interest

There are no conflicts to declare.

## Data Availability

The datasets generated and analyzed during the current study were obtained from first-principles density functional theory (DFT) calculations performed using the WIEN2k package. The electronic structure, magnetic, and optical properties were calculated using WIEN2k within the full-potential linearized augmented plane wave (FP-LAPW) method, with spin–orbit coupling (SOC) treated self-consistently where relevant. Convergence criteria were based on total energy, total charge, and atomic forces. The thermoelectric transport properties were computed using the BoltzTraP code interfaced with WIEN2k within the constant relaxation-time approximation. All relevant input and output files—including structural (.struct), electronic (.in1, *.in2, *.inorb, .inc), spin–orbit coupling, optical (.inop, *.joint), magnetic, and BoltzTraP input/output files—as well as supporting results such as band structures, density of states, optical spectra, and thermoelectric properties (Seebeck coefficient and *ZT*), are available from the corresponding author upon reasonable request.
